# Intrinsic Risk Factors for Ankle Sprain Differ Between Male and Female Athletes: A Systematic Review and Meta-Analysis

**DOI:** 10.1186/s40798-022-00530-y

**Published:** 2022-11-18

**Authors:** Joel Mason, Christoph Kniewasser, Karsten Hollander, Astrid Zech

**Affiliations:** 1 Department of Human Movement Science and Exercise Physiology, Friedrich Schiller University Jena, Seidelstraße 20, 07749 Jena, Germany; 2 Institute of Interdisciplinary Exercise Science and Sports Medicine, Medical School Hamburg, Hamburg, Germany

**Keywords:** Injury, Prognostic, Sex-specific, Women, Men, Sport, Strength, Balance

## Abstract

**Background:**

Ankle sprains remain prevalent across most team sports. However, despite divergent ankle sprain injury rates in male and female athletes, little is known about potential sex-specific risk factors for ankle sprain.

**Objective:**

To systematically investigate the sex-specific risk factors for ankle sprain.

**Methods:**

Combinations of the key terms were entered into PubMed, Web of Science, Embase and Cochrane Library databases, and prospective studies reporting ankle sprain risk factors in males or females were included for meta-analysis.

**Results:**

Sixteen studies were eligible for inclusion, for a total of 3636 athletes (735 female) and 576 ankle sprains (117 female). Out of 21 prognostic factors, previous ankle sprain injury (odds ratio = 2.74, *P* < .001), higher body mass index (SMD = 0.50, *P* < 0.001), higher weight (SMD = 0.24, *P* = 0.02), lower isometric hip abduction strength (SMD = − 0.52, *P* < 0.0001) and lower dynamic balance performance (SMD = − 0.48 to − 0.22, *P* < 0.001–0.04) were identified as risk factors in male athletes. In female athletes, out of 18 factors eligible for meta-analysis, only lower concentric dorsiflexion strength was identified as a risk factor (SMD = − 0.48, *P* = 0.005).

**Conclusion:**

This meta-analysis provides novel evidence for different risk factor profiles for ankle sprain injuries between female and male athletes. Further studies, particularly in female athletes, are needed to strengthen the evidence.

**Supplementary Information:**

The online version contains supplementary material available at 10.1186/s40798-022-00530-y.

## Key Points

1. Risk factors for ankle sprain in male athletes include a previous ankle sprain, higher weight and body mass index, poor dynamic balance performance and lower hip strength.
2. Lower specific ankle strength was the only risk factor identified for ankle sprains in female athletes.
3. There are different risk factors for ankle sprain in males and females, but a shortage of data specifically in female athletes prevents stronger conclusions from being drawn.

## Introduction

Ankle sprains are prevalent across a broad range of sports, accounting for up to 22.6% of all injuries in American collegiate and high school athletes [1, 2]. Incidence rates are particularly high in sports which involve large volumes of running, change of direction and jumping/landing [3, 4], evidenced by just over one quarter of National Basketball Association players sustaining an ankle sprain each season [5]. This high prevalence is exacerbated by the frequency of reinjury following an initial ankle sprain, with studies observing that up to 47% of injuries were recurrent [6].

Due to their prominence in the sports injury landscape and the tendency for ongoing complications post-injury [7, 8], the prevention of ankle sprains is paramount. While external interventions such as ankle braces have proven both convenient and effective [9, 10], the characterisation of intrinsic risk factors predisposing an athlete to injury forms a critical stage of the injury prevention process and ultimately empowers practitioners to develop targeted strategies to reduce injury risk which may also benefit long-term athletic development [11–14]. Aside from the widely reported higher injury risk associated with a previous ankle sprain [15–18], deficits in muscular strength, proprioception, dynamic balance performance and co-ordination, as well as higher or lower body mass index (BMI) have all been reported to heighten the risk of sustaining an ankle sprain [10, 17, 19–21].

Biological sex is also considered a risk factor for ankle sprain. Although there are reports of comparable incidence rates between males and females [22], a 2014 systematic review concluded that females suffer from ankle sprains at higher rates than their male counterparts [23], which aligns with recent evidence indicating sex-specific general injury patterns in team sports [24] and running [25]. Sex-based differences in factors such as joint laxity and sensorimotor control may contribute to this injury rate discrepancy [26, 27], and there is early evidence that ankle sprain injury history influences future ankle sprain risk in males but not females [15]. However, very few studies have directly compared ankle sprain risk factors between sexes, and aggregated data approaches obscure potential sex-based differences. This uncertainty regarding the extent to which the risk factors for ankle sprain differ between males and females may inhibit the development of more targeted and effective strategies to mitigate injury risk. Therefore, the objective of this study was to generate a summary from the available evidence through a meta-analysis identifying the intrinsic risk factors for ankle sprain in male and female athletes.

## Methods

This systematic review and meta-analysis were conducted in accordance with the PRISMA guidelines for reporting systematic reviews and meta-analyses [28].

### Search Strategy

One investigator (JM) conducted a systematic literature search of articles published between January 2000 and September 2021 using PubMed, Web of Science, Embase and Cochrane Library databases. For these, the terms “ankle sprain” OR “ankle injury” were combined (AND) with “risk*” OR “predict*”. All identified article titles and abstracts were then exported to Rayyan [29]. Within Rayyan, two authors (JM and AZ) screened articles for eligibility firstly according to title and abstract and the remaining articles had their full text screened. The bibliographical information of included articles was also examined for further relevant articles.

### Eligibility Criteria

At the title and abstract screening stage, publications were considered relevant if they (1) included a prospective design, (2) demonstrated visible documentation of ankle injury, (3) included reporting of at least one risk factor or prognostic factor and (4) were published in an English peer-reviewed journal. Studies were excluded if they were related to other types of injury rather than ankle sprain, if they were reviews, if they clearly included populations that were not involved in structured sport participation, or if they did not investigate risk factors. During title and abstract screening, if insufficient information was available to determine study eligibility, the study was passed through to the full-text screening stage for further inspection.

The eligibility criteria used at the full-text screening stage were similar. Articles were screened for inclusion based on the following criteria: (1) prospective cohort design, (2) included ankle sprain injury data, (3) reported at least one potential internal risk factor for ankle sprain injury, (4) reported specific and separate data for male or female athletes (or both separately), (5) published in the English language, (6) publication in a peer-reviewed journal, (7) reporting of data specifically on ankle sprains rather than general ankle injury or general injury, (8) included participants involved in structured sport participation. Ankle sprain injuries were defined as injuries prohibiting an athlete from full participation in training for a minimum of one day. Studies which included active participants who were not necessarily involved in structured sport (for example, those in military populations), retrospective studies, studies which used an intervention, studies which did not provide data for males and/or females separately and studies which did not provide separate data for injured and uninjured groups were ruled ineligible for inclusion. In the event of conflict between the two reviewers regarding eligibility of articles, a third reviewer (CK) was utilised to form a majority decision.

### Data Extraction

Study characteristics such as sex, observation period, sport type, level of play and sample size were extracted. The number of ankle sprain injuries and the rate of injuries per season were extracted (outcome measurements), as well as the potential risk factors (prognostic measurements). The prognostic measurements were included with mean and standard deviation. For studies that reported the mean and standard error, this was converted to standard deviation. Dichotomous variables were included using the raw data. Units reported in anything but metric units were subsequently converted to metric units to allow meta-analysis. All data extraction was completed by one author (CK).

### Study Quality and Risk of Bias Assessment

The risk of bias assessment was undertaken by two reviewers separately (CK and JM) using a modified version of the “Quality in Prognosis Studies” (QUIPS) tool [30, 31]. The QUIPS uses six different categories to measure the risk of bias. The category “study participation” is used to assess the risk of selection bias. For this purpose, 5 questions are asked which must be answered with either yes or no. “Study attrition”, “prognostic outcome measurement”, “outcome measurement”, “study confounding” and “statistical analysis and reporting” form the remaining 5 categories. Here also several questions are asked, which must be answered with yes or no. To fulfill a low risk in a category, at least 75% of the questions must be answered with yes. To be rated as a low-risk study, at least 5 low-risk categories must be achieved. The “outcome measurement” category also has a special role. Since this category has only three questions, all of them must be answered with yes and to be marked as a low-risk study, this category must be fulfilled. The QUIPS has been previously described [30, 31] and was also used by previous risk factor reviews [32–35].

### Statistical Analysis

The meta-analysis was performed using Review Manager (version 5.4.1) [36] and included all risk factors with more than one study in females and males. Continuous data of the prognostic measurements were converted into standardised mean differences (SMD) and 95% confidence intervals, with the SMD reflecting the magnitude of the difference between injured and uninjured athletes. For dichotomous variables, the raw data were analysed with the method of Mantel–Haenszel, and the effect measure was reported as odds ratio and 95% confidence intervals. Data were pooled between studies using a random effects model [37] and summarised in a forest plot. This was chosen because it more conservatively estimates effect sizes and mitigates potential methodological differences and statistical heterogeneity. *Z* statistics and *P*-values were calculated to assess if the effect was statistically significant. Statistical significance was set at *P* ≤ 0.05. Heterogeneity was assessed using the *I*^2^ statistics, with a higher value indicating a higher heterogeneity [38].

## Results

### Search Results

**Fig. 1:**
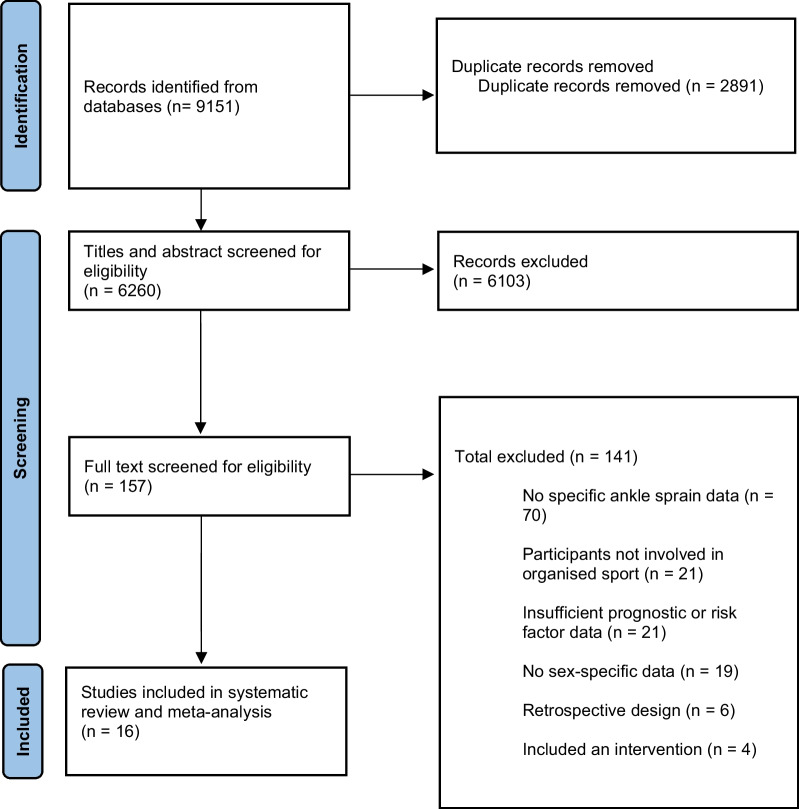
PRISMA flow diagram of the identification and selection of the studies included in this meta-analysis. PRISMA: Preferred Reporting Items for Systematic Reviews and Meta-Analyses

The systematic search yielded 9151 articles. After removal of duplicates, 6260 articles remained. A total of 157 articles were included for full-text analysis after selection of titles and abstracts. Of these 157, 70 were excluded due to a lack of specific ankle sprain injury data, 21 were removed due to their participants not being involved in organised sport, 21 were excluded for insufficient prognostic or risk factor data, 19 were removed due to not providing sex-specific data, 6 were excluded due to a retrospective design and 4 were eliminated due to using an intervention, leading to an overall exclusion of 141 manuscripts at this stage of screening. Therefore 16 articles were ultimately able to satisfy the inclusion criteria of the meta-analysis (Fig. 1) [16–21, 39–48]. These included studies were published between 2001 and 2021.

### Description of the Included Studies

Table 1 provides details of the included studies. The eligible studies captured 576 ankle sprains (male 459, female 117) in a pool of 3636 athletes (male 2901, female 735). A total of 2079 of these athletes participated at an amateur level of sport (57.2%), 745 at a subelite level (20.5%) and 812 on an elite level (22.3%), as self-described by the studies. The rate of ankle sprains per season was similar in males (11.5%) and females (11.4%).

**Table 1:** Descriptions of included studies

Study	Sex	Sample	Level	Ankle sprains	Prognostic measurements	Length of tracking
Attenborough et al. 2017 [39]	Female	94 netball players	Subelite, amateur	11	**Age, Height, Weight, Injury history, SEBT (dynamic balance),** CMJ, inversion–eversion (ankle joint laxity), CAIT-Y (joint instability), demi- pointe test (static balance), foot lift test (static balance), level of play	1 season
Beynnon et al. 2001[40]	Female and male	68 female and 50 male college athletes from various sports	Subelite	f: 13 m: 7	**Weight,** Height**, Dorsiflexion ROM, anterior drawer (ankle joint laxity)**, **concentric strength (plantarflexion, dorsiflexion, inversion, eversion)**, **eccentric strength**(plantarflexion, dorsiflexion, **inversion, eversion**)leg dominance, modified Beighton method (generalszed joint laxity), anatomic foot type, **inversion ROM** (unweighted), **eversion ROM** (unweighted), rearfoot varus/valgus ROM (unweighted), forefoot/rearfoot relationship, Dorsiflexion ROM (knee extended, weightbearing), calcaneus varus/valgus (weightbearing), tibial varus valgus (weightbearing), concentric and eccentric ratio (dorsiflexion/plantarflexion, eversion/inversion), A-P centre of gravity sway (static balance)	2 seasons
DeRidder et al. 2016 [41]	Male	133 youth soccer players	Subelite	12	**Age, BMI, isometric strength** (hip flexion, extension, **abduction**, adduction, external rotation, internal rotation), playing experience, leg dominance, adjusted body size, exposure	3 seasons
Engebretsen et al. 2010 [42]	Male	508 professional soccer players	Elite	43	**Age, Weight, Height, BMI, Injury history, anterior drawer (ankle joint laxity)**, **Dorsiflexion ROM** FAOS function score, single leg balance test (static balance), clinical examination, foot type, standing rearfoot alignment, hallux position, supination ROM, pronation ROM, playing position	1 season
Fousekis et al. 2012 [43]	Male	100 professional soccer players	Elite	17	**Age, Weight, Height, BMI, anterior drawer (ankle joint laxity),** concentric and eccentric strength (dorsiflexion and plantarflexion) dorsiflexion ROM, plantarflexion ROM, lower limb functional length, tibia length, proprioception (all not included parameters are dichotomous)	1 season
Gribble et al. 2015 [21]	Male	539 high school and college American football athletes	Amateur	54	**Age, Weight, Height, BMI, Injury history, SEBT (dynamic balance),** modified FMS	1 season
Hartley et al. 2018 [20]	Female and male	167 female and 384 male collegiate athletes from various sports	Subelite	f: 21 m: 38	**BMI, Injury history, YBT (dynamic balance),** YBT asymmetry, **Dorsiflexion ROM,** modified BESS (static balance)	2 seasons
Kawaguchi et al. 2021 [44]	Male	145 collegiate soccer players	Subelite	31	**Age, Weight, Height, BMI, Injury history, Dorsiflexion ROM,** Playing experience, muscle mass, fat mass, general joint laxity, bilateral balance test (force plate), unilateral balance test (force plate), height of navicular tubercle, knee extension ROM, hip internal rotation ROM, muscle flexibility (Iliopsoas, quadriceps, hamstring, gastrocnemius, soleus), **isometric strength** (knee extension and flexion, **hip abduction**)	1 season
Kofotolis et al. 2007 [18]	Male	312 soccer players	Amateur	139	**Injury history**	2 seasons
Kofotolis et al. 2007 [45]	Female	204 professional basketball players	Elite	32	**Injury history**	2 seasons
McCann et al. 2018 [46]	Female	43 collegiate soccer athletes	Subelite	8	**Weight, Height, BMI, Injury history**	1 season
Powers et al. 2017 [19]	Male	185 youth and adult professional soccer players	Subelite, elite	25	**Age, Weight, Height, BMI, Injury history, isometric strength (hip abduction)**	1 season
Saki et al. 2021 [16]	Male	152 youth athletes from various sports	Amateur	34	**Age, Weight, Height, BMI, Injury history, Dorsiflexion ROM, Plantarflexion ROM,** navicular drop, Q angle, tibia vara, tibia rotation, knee recurvatum	2 seasons
Tyler et al. 2006 [17]	Male	152 American football high-school athletes	Amateur	15	**Injury history,** BMI (dichotomous)	1–3 seasons
Willems et al. 2005 [47]	Male	241 sport students	Amateur	44	**Age, Weight, Height, BMI, Dorsiflexion ROM, Plantarflexion ROM,** joint position sense, flamingo balance, standing broad jump, shuttle run, endurance shuttle, concentric and eccentric strength (inversion, eversion, plantarflexion, dorsiflexion), **Inversion ROM**, **Eversion ROM**, metatarsophalangeal-1-joint flexion ROM, metatarsophalangeal-1-joint extension ROM, hip external rotation ROM, hip internal rotation ROM, calcaneus position subtalar joint in neutral position (weighted, unweighted, and weighted with subtalar joint not in neutral position), postural control (force-plate), muscle reaction time (peroneus longus, peroneus brevis, tibialis anterior, gastrocnemius)	1 season
Willems et al. 2005 [48]	Female	159 sport students	Amateur	32	**Age, Weight, Height, BMI, Dorsiflexion ROM**, **Plantarflexion ROM**, joint position sense, concentric and eccentric strength (inversion, eversion, plantarflexion, dorsiflexion), **Inversion ROM**, Eversion ROM, metatarsophalangeal-1-joint flexion ROM, metatarsophalangeal-1-joint extension ROM, hip external rotation ROM, hip internal rotation ROM, calcaneus position subtalar joint in neutral position (weighted, unweighted, and weighted with subtalar joint not in neutral position), postural control (force-plate)	1 season

*SEBT* Star excursion balance test, *CMJ* Counter movement jump, *CAIT-Y* Cumberland ankle instability tool-youth, *f* Female, *M* Male, *ROM* Range of motion, *A-P* Anterior–posterior, *BMI* Body mass index, *FAOS* Foot and ankle outcome score, *FMS* Functional movement screen, *YBT* Y-Balance Test, *BESS* Balance error scoring system, *Q angle* Quadriceps angle. Bold text denotes outcomes that were eligible for meta-analysis

There were 10 studies investigating males only, 4 investigating females only and two studies investigating both sexes separately. The age range for males was 10–34, whereas the age range for included females was 17–26.

The included male population came from soccer (52.5%), American football (29.4%), multisport (8.69%), volleyball (2.9%), basketball (2.7%), baseball (1.9%), lacrosse (1.5%), tennis (0.3%) and handball (0.2%). The included female population came from basketball (32.5%), multisport (24.9%), soccer (15%), netball (12.8%), field hockey (4.2%), softball (4.1%), volleyball (4.1%) and lacrosse (2.5%).

Six studies reported any ankle sprain [18, 20, 32, 39, 40, 45], six studies reported lateral ankle sprains only [21, 41, 44, 46–48], three studies reported non-contact ankle sprains only [17, 19, 43], and one study investigated non-contact lateral ankle sprains only [16].

For the studies that reported the complete exposure hours, the ankle sprain incidence rates ranged from 0.75 to 1.74 injures per 1000 exposure hours in females and 0.36–2.17 injuries per 1000 exposure hours in males.

### Overview of Results of Risk of Bias Assessment (QUIPS)

Risk of bias assessment details is provided in Table 2. In general, a low risk of bias was detected for studies included in this review. Specifically, low risk of bias was found in 11 studies (68.75%) [18–21, 40–46] and high risk of bias in 5 studies (31.25%) [16, 17, 39, 47, 48]. The authors were able to reach complete consensus in the assessment of risk of bias. “Study confounding” was most frequently identified as a potential source of bias (37.5% of all studies), followed by “outcome measurement” (31.25%), “study attrition” (25%) and “study participation” (6.25%). The items “prognostic factor measurement” and “statistical analysis and reporting” were met by all studies.

**Table 2:** QUIPS risk of bias assessment for included studies

Study	Potential risk of bias item						Risk of bias
	1	2	3	4	5	6	
Attenborough et al. 2017 [39]	+	+	+	−	−	+	High
Beynnon et al. 2001 [40]	+	+	+	+	−	+	Low
DeRidder et al. 2016 [41]	+	+	+	+	+	+	Low
Engebretsen et al. 2010 [42]	+	+	+	+	+	+	Low
Fousekis et al. 2012 [43]	+	+	+	+	+	+	Low
Gribble et al. 2016 [21]	+	+	+	+	+	+	Low
Hartley et al. 2018 [20]	+	+	+	+	+	+	Low
Kawaguchi et al. 2021 [44]	+	+	+	+	+	+	Low
Kofotolis et al. 2007 [18]	+	−	+	+	+	+	Low
Kofotolis et al. 2007 [45]	+	−	+	+	+	+	Low
McCann et al. 2018 [46]	+	+	+	+	−	+	Low
Powers et al. 2017 [19]	+	−	+	+	+	+	Low
Saki et al. 2021 [16]	+	−	+	−	+	+	High
Tyler et al. 2006 [17]	−	+	+	−	−	+	High
Willems et al. 2005 [47]	+	+	+	−	−	+	High
Willems et al. 2005 [48]	+	+	+	−	−	+	High

*QUIPS* Quality in Prognosis Studies. 1: study participation, 2: study attrition, 3: prognostic factor measurement, 4: outcome measurement, 5: study confounding variables, 6: statistical analysis and reporting

### Overview of Results from Meta-Analysis

**Fig. 2:**
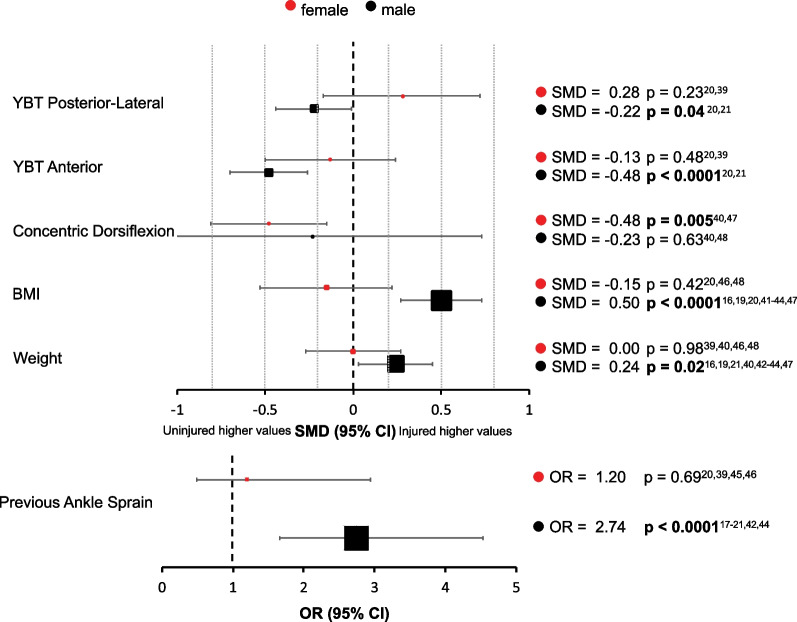
Summary forest plot including variables identified as risk factors for ankle sprain in males and females, with standardised mean difference or odds ratio and 95% confidence intervals. The size of the marker represents the sample size (larger marker = larger subject pool). *YBT* Y-Balance Test, *BMI* body mass index, *OR* odds ratio

A total of 21 risk factors could be included in the meta-analysis, of which 17 factors allowed sex comparison. Isometric hip abduction, plantarflexion ROM, hip internal rotation and anterior drawer test for ankle could only be analysed for the male population due to an absence of studies in females. Risk factors were subsequently classified into athlete characteristics (including age, height, weight, BMI and ankle sprain injury history), strength, dynamic balance, joint range of motion and joint laxity. For the male population, previous injury, higher BMI and weight, deficient Y-Balance Test (YBT) anterior reach and posterior-lateral reach distance and poor isometric hip abduction strength were identified as risk factors for ankle sprain injury, whereas in the female population, only concentric dorsiflexion strength was shown to be a significant risk factor (Fig. 2). Forest plots for all risk factors are available in Additional file 1.

### Athlete Characteristics

For male athletes, the meta-analysis revealed an association between higher weight (SMD = 0.24, 95% CI 0.03–0.45, *P* = 0.02, *I*^2^ = 56%) [16, 19, 21, 40, 42–44, 47], higher BMI (SMD = 0.50, 95% CI 0.27–0.73, *P* < 0.0001, *I*^2^ = 69%) [16, 19, 21, 40, 42–44, 47], a previously injured ankle (OR = 2.74, 95% CI 1.66–4.53, *P* < 0.0001, *I*^2^ = 72%) [17–21, 42, 44] and the occurrence of an ankle sprain. Age (SMD = 0.19, 95% CI − 0.06–0.43, *I*^2^ = 69%) [16–21, 41–44, 47] and height (SMD = − 0.07 95% CI − 0.45–0.32, *I*^2^ = 87%) [16, 19, 21, 42–44, 47] were not associated with sustaining an ankle sprain in males. No athlete characteristic risk factor (age[ SMD = − 0.23 95% CI − 0.56 to − 0.10, *I*^2^ = 0%] [39, 48], weight [SMD = − 0.00 95% CI − 0.27–0.27, *I*^2^ = 0%] [39, 40, 46, 48], height [SMD = 0.14 95% CI − 0.34–0.62, *I*^2^ = 51%] [39, 46, 48], BMI [SMD = − 0.15 95% CI − 0.53–0.22, *I*^2^ = 39%] [20, 46, 48], previous ankle sprain[OR = 1.20 95% CI 0.49–2.94, *I*^2^ = 60%] [20, 35, 39, 46]) had an influence on the occurrence of an ankle sprain in female athletes.

### Strength

Reduced concentric dorsiflexion strength in females (SMD = − 0.48, 95% CI − 0.81 to − 0.15, *P* = 0.005, *I*^2^ = 0%) [40, 48] and reduced isometric hip abduction in males (SMD = − 0.52, 95% CI − 0.78 to − 0.26, *P* < 0.0001, *I*^2^ = 0%) [19, 41, 44] were associated with an increased risk of ankle sprain. It should also be noted that no studies investigated the influence of isometric hip abduction strength on ankle sprain incidence in females. The other strength parameters revealed no association with an ankle sprain, either in male (concentric plantarflexion [SMD = − 0.09 95% CI − 0.85–0.67, *I*^2^ = 69%] [40, 47], dorsiflexion [SMD = − 0.23 95% CI − 1.20–0.73, *I*^2^ = 80%] [40, 47], inversion [SMD = − 0.14 95% CI − 0.44–0.17, *I*^2^ = 0%] [40, 47] and eversion [SMD = 0.21 95% CI − 0.09–0.51, *I*^2^ = 0%] [40, 47]; eccentric inversion [SMD = − 0.08 95% CI − 0.45–0.28, *I*^2^ = 14%] [40, 47] and eversion [SMD = 0.14 95% CI − 0.16–0.45, *I*^2^ = 0%] [40, 47]), or in female athletes (concentric plantarflexion [SMD = − 0.13 95% CI − 0.46–0.19, *I*^2^ = 0%] [40, 48], inversion [SMD = − 0.03 95% CI − 0.36–0.29, *I*^2^ = 0%] [40, 48] and eversion [SMD = − 0.21 95% CI − 0.54–0.11, *I*^2^ = 0%] [40, 48]; eccentric inversion [SMD = − 0.08 95% CI − 0.59–0.42, *I*^2^ = 51%] [40, 48] and eversion [SMD = − 0.12 95% CI − 0.45–0.21, *I*^2^ = 0%] [40, 48]).

### Dynamic Balance

Reduced Y-Balance Test anterior reach distance (SMD = − 0.48, 95% CI − 0.70 to − 0.26, *P* < 0.0001, *I*^2^ = 0%) [20, 21] and posterior-lateral reach distance (SMD = − 0.22, 95% CI − 0.44 to − 0.01, *P* = 0.04, *I*^2^ = 0%) [20, 21] in the male athletes were associated with the occurrence of ankle sprain. Posterior-medial reach (SMD = − 0.04 95% CI − 0.26–0.17, *I*^2^ = 0%) [20, 21] distance had no influence on injury risk. For female athletes, no difference in the injured and the uninjured population was observed in the YBT (anterior [SMD = − 0.13 95% CI − 0.50–0.24, *I*^2^ = 0%] [20, 39], posterior-lateral [SMD = 0.28 95% CI − 0.17–0.72, *I*^2^ = 28%] [20, 39], posterior-medial [SMD = 0.15 95% CI − 0.51–0.82, *I*^2^ = 66%] [20, 39]).

### Range of Motion and Joint Laxity

Ankle joint laxity in the form of the ankle anterior drawer test did not reveal an association with higher risk of ankle sprain in male athletes (OR = 1.49 95% CI 0.83–2.69, *I*^2^ = 0%) [40, 42, 43]. No factor related to joint range of motion showed a relationship with risk of ankle sprain in male (dorsiflexion [SMD = − 0.03 95% CI − 0.56 to − 0.10, *I*^2^ = 0%] [16, 20, 40, 42, 44, 47], plantarflexion [SMD = − 0.28 95% CI − 0.76–0.20, *I*^2^ = 73%] [16, 47], inversion [SMD = − 0.07 95% CI − 0.54–0.40, *I*^2^ = 34%] [40, 47], eversion [SMD = 0.16 95% CI − 0.59–0.91, *I*^2^ = 68%] [40, 47] and hip internal rotation [SMD = − 0.03 95% CI − 0.30–0.24, *I*^2^ = 16%] [44, 47]) or female athletes (dorsiflexion [SMD = 0.12 95% CI − 0.15–0.38, *I*^2^ = 0%] [20, 40, 48], inversion [SMD = − 0.04 95% CI − 0.36–0.29, *I*^2^ = 0%] [40, 48], eversion [SMD = 0.17 95% CI − 0.24–0.57, *I*^2^ = 28%] [40, 48].

## Discussion

This is the first study to systematically identify sex differences in intrinsic risk factors for ankle sprain in athletes using a meta-analytic approach, with the major finding that different risk factors have been reported for males and females. Males with a previous ankle sprain, higher weight and body mass index, lower isometric hip abduction strength and worse performance in multiple dynamic balance directions were identified as having an elevated risk for ankle sprains. However, only females with lower concentric dorsiflexion strength were detected to be at a higher risk of sustaining an ankle sprain. Our findings not only preliminarily suggest divergent risk factors between males and females, but also highlight a clear paucity of data regarding female-specific risk factors for ankle sprain injury in athletes. Only 20.2% of participants in our analyses were female, which likely contributed to a lack of evidence for further risk factors for female athletes. Therefore, it cannot be ruled out that there are other under-researched factors contributing to ankle sprain risk in female athletes.

A key finding of this study is that previous ankle sprain was identified as a leading risk factor for future ankle sprain in male athletes but not in female athletes, which is supported by Wikstrom and colleagues [15]. Evidence indicates that those with a history of ankle sprain display alterations in central processing which purportedly feed into future ankle sprain injury risk. These alterations include differences in visual processing during single leg tasks [49], shifts in muscular activation strategies during landing tasks [50], and changes in muscle activation patterns in both the injured and healthy limbs during perturbed walking tasks [51]. However, given that these findings are from mixed-sex studies, it remains unclear why ankle sprain history is a risk factor for males but not females. Considering findings that ankle sprain patients often develop prolonged changes in ankle joint laxity and local muscle weakness, it is conceivable that deficient local ankle muscle strength would play a role in increased vulnerability for subsequent ankle sprain risk in male athletes [52, 53]. However, contrary to this notion is that no significant relationship between local ankle strength and ankle sprains was detected in males in our study and instead, female athletes with deficient concentric dorsiflexion strength were more likely to sustain an ankle sprain. Although mixed-sex studies report lower dorsiflexion strength in people with a history of ankle sprain [54], our results indicate that females with deficits in concentric dorsiflexion strength are at a higher risk of ankle sprain regardless of injury history and that dorsiflexion strength should therefore be considered an independent risk factor.

Although local ankle muscle strength was not significantly associated with ankle sprain risk in male athletes, we identified an association between global strength and ankle sprains, with male athletes demonstrating lower isometric hip abduction strength at a higher risk of injury. In support of this finding is mixed-sex evidence suggesting that people with hip-abductor weakness exhibit altered ankle mechanics during single leg landing and balance tasks [55, 56]. Further, males and females with a history of ankle sprain demonstrate altered hip muscle activation strategies during landing under fatigued conditions [50], as well as lower hip abduction strength than healthy controls [57]. This may further explain identification of a previous ankle sprain as a risk factor for a future ankle sprain injury in men. However, despite the mechanistic evidence which links hip function and factors relevant for ankle sprain risk in mixed-sex studies, the relationship between isometric hip abduction strength and ankle sprain in females remains unclear due to a complete absence of studies. We therefore recommend that future studies investigate the role of isometric hip abduction strength in ankle sprain incidence specifically in females.

Our analysis also revealed that deficient dynamic balance performance in multiple directions was a risk factor for ankle sprain in males but not females. In pooled-sex studies, deficits in dynamic balance performance persist at least six months following an initial injury [58], and those with a history of ankle sprain also experience greater decrements in dynamic balance performance under fatigued conditions than healthy controls [59]. This may underpin the association between ankle sprain history and future ankle sprain risk in male athletes. Females typically score higher than males on tests commonly used to assess dynamic balance [60], and female athletes experience no significant decrements in Star excursion balance test performance following a whole-body fatiguing protocol [61] which may help to explain the lack of relationship between dynamic balance and ankle sprain risk in female athletes. Despite neuromuscular training programmes enhancing dynamic balance performance [62], our finding that dynamic balance has no influence on ankle sprain incidence in female athletes may also elucidate the inconclusive effects of neuromuscular training programmes on ankle injury prevention in female soccer players [63]. This is supported by evidence showing that neuromuscular training incorporating only balance exercises is less effective for preventing ankle sprains than multimodal exercise programmes using a combination of balance, strength or stretching exercises [64]. Further, there are inconclusive findings regarding whether or not balance training can improve other neuromuscular parameters [65], and therefore training designed to mitigate the risk of ankle sprain in females is recommended to look beyond only balance and address many components of neuromuscular development. For example, although not eligible for meta-analyses due to a lack of studies, there is isolated evidence that deficits in joint position sense during ankle inversion (considered an indicator of proprioception) and poor co-ordination also influence ankle sprain risk in females [48]. Further, given recent findings that postural control under fatigued conditions is also altered in the premenstrual phase for female athletes [66], combined with increasing evidence for hormonal influences on anterior cruciate ligament injury risk in female athletes [67, 68], interactions between neuromuscular control, menstrual cycle phase and ankle sprain injury risk warrant further investigation.

Finally, both weight and BMI were associated with ankle sprain incidence rate in male but not female athletes. Importantly, these risk factors likely interact with other risk factors to jointly influence the risk of ankle sprain injury in male athletes. Tyler et al. [17] observed that an overweight high school American football player with a history of ankle sprain was 19 times more likely to sustain an ankle sprain than a player who was within a normal weight range and had no history of ankle sprain. Indeed, risk factors that are independently identified likely interact to influence injury risk in a joint fashion [69, 70], as highlighted throughout this discussion. Further evidence of this comes again from the male-specific risk factors in the current study, with De Ridder and colleagues [41] suggesting that decreases in hip strength may contribute to reduced dynamic control of the hip joint, which likely influences performance on dynamic balance tests [71].

Overall, previous studies on movement-related mechanisms contributing to sports injuries have often neglected or underestimated the influence of female vs. male characteristics. Therefore, the reasons for the different injury risk factors between males and females are not fully understood. Sex differences have been observed for jump [72] and jump landing biomechanics [73] as well as change of direction [74], squatting and side-step tasks [75], indicating different strategies of neuromuscular control to stabilise the ankle joint during challenging movements. This is supported by our finding that dorsiflexion strength is associated with ankle sprain risk in females but not in males. Another factor that has often been discussed in relation to the injury risk in females is a greater joint laxity and lower joint resistance to translation and rotation movements when compared with males [26, 76]. However, in our meta-analysis no factor related to joint range of motion showed a relationship with risk of ankle sprain in both females and males.

It would be negligent to not comment on a central issue which is not only a limitation of our study, but more importantly, a shortcoming of the ankle sprain and sport science literature in general: the presence of systematic bias resulting in a distinct lack of female-specific research [77, 78]. In the case of our analysis, this gap is further highlighted by the pooled sample sizes. Of the 3636 athletes, only 20.2% were females, and of the 576 ankle sprains, only 20.3% occurred in females. These percentages are even comfortably below the recent revelations that between 34 and 39% of participants in sports and exercise medicine and science research are females [77, 78]. Such disparities in sample sizes between sexes may have further contributed to the absence of risk factors found for female ankle sprains in our study and ultimately limit our ability to make strong conclusions regarding female-specific risk factors for ankle sprain. Indeed, when considered alongside the evidence that females experience ankle sprains at higher rates, our finding of fewer risk factors for ankle sprain in women highlights the insufficiency of current data.

Importantly, our analysis also highlights the hazards of pooling sexes to interpret results. When studies were combined for sexes in the current study, six variables were detected as risk factors for ankle sprain, but when males were removed from the analysis, only one risk factor remained significant for females. This result has important implications for the interpretation of findings from sports injury studies, indicating that findings from male and mixed-sex studies should not simply be extrapolated and applied to females without due consideration.

### Limitations

A central limitation of this study has been outlined in the preceding paragraphs, and the authors emphasise that further female-specific data are needed to make stronger conclusions regarding whether mechanisms or methodology underlie the observed sex-differences in ankle sprain risk factors. Additionally, in the same way that studies pooling results for sexes limit insight into the sex-dependent risk factors for ankle sprain, our approach to combining studies likely masks sport-dependent, age-dependent and level-dependent risk factors for ankle sprain injury risk. It is certainly conceivable that elite basketballers with well-developed physical capacities demonstrate different risk factors for ankle sprain than adolescent amateur soccer players, and there is ample existing evidence for different ankle sprain injury rates between sports [3, 79]. We also recognise that different types of ankle sprain injury and different mechanisms of injury likely have unique risk factors, and we therefore acknowledge this as a limitation of our study. The methodological heterogeneity should also be considered when interpreting our results, particularly for risk factors where the *I*^2^ value is high, such as male concentric dorsiflexion strength (*I*^2^ = 80%).

### Recommendations

The results of this analysis provide practitioners with clear targets for reducing ankle sprain injury in male athletes. Data on previous injury history should be considered in all cases, and all interventions should target the development of isometric hip abduction strength and dynamic balance performance, alongside potential reductions in BMI in specific cases. Indeed, there is evidence that a balance training intervention can drastically reduce the elevated injury risk that arises from ankle sprain history and a higher BMI in male high school American football players [80]. Further, although outside the scope of this study, external ankle support appears to improve ankle sprain avoidance and reoccurrence outcomes [81, 82]. The absence of multiple risk factors in our study should not discourage practitioners from performing injury risk screening and implementing injury prevention programmes in female athletes. We acknowledge and emphasise that our findings do not entirely dismiss some risk factors due to issues arising from small sample sizes and low quality of evidence. We therefore suggest that the development of concentric dorsiflexion strength should be considered an integral component of screening and interventions and should be implemented as part of a wider neuromuscular training programme in order to mitigate the risk of ankle sprain injury in female athletes. As it is plausible that different types of ankle sprain have different risk factors, we recommend that future studies seek to identify ankle sprain injury type-specific risk factors and elucidate the mechanisms underlying specific injury risk factors in order to provide practitioners with more actionable information. For example, it is currently unclear why dorsiflexion strength is associated with ankle sprain injury in females but not males.

### Conclusion

These results provide the first meta-analytic evidence that male and female athletes may have unique risk factors for ankle sprains. However, the strength of this conclusion is somewhat limited due to methodological considerations, and the risk factors which drive higher ankle sprain injury rates in female athletes remain largely elusive based on the available evidence. We therefore encourage future studies to disaggregate their data according to sex wherever possible, as well as to seek further elucidation of the contributions of isometric hip abduction strength, joint laxity and injury history to ankle sprain incidence specifically in female athletes.

## Supplementary Information

**Additional file 1.** Forest plots for potential risk factors for ankle sprain in males and female athletes.

## Data Availability

The data from the current review are presented in the article/electronic supplementary material and are available from the corresponding author upon request.
