# Supplementary material for: Intrinsic Risk Factors for Ankle Sprain Differ Between Male and Female Athletes: A Systematic Review and Meta-Analysis
Source: Sports Med Open. 2022 Nov 18;8:139. doi: 10.1186/s40798-022-00530-y (PMC9674823; doi:10.1186/s40798-022-00530-y)
Supplement: Supplementary file 1 — Additional file 1. Forest plots for potential risk factors for ankle sprain in males and female athletes. [file 40798_2022_530_MOESM1_ESM.pdf]

Supplementary File 1:

Intrinsic risk factors for ankle sprain differ between male and female athletes: a systematic review and meta-analysis

Sports Medicine Open

Joel Mason[1], Christoph Kniewasser[1], Karsten Hollander[2] & Astrid Zech[1]

[1]Department of Human Movement Science and Exercise Physiology, Friedrich Schiller University Jena, Jena 07749, Germany

[2]Institute of Interdisciplinary Exercise Science and Sports Medicine, Medical School Hamburg, Hamburg, Germany

Corresponding author contact details:

Joel Mason, PhD

Seidelstraße 20, 07749 Jena, Germany

Joel.Mason@uni-jena.de

Study or Subgroup	Experimental Mean	Experimental SD	Experimental Total	Control Mean	Control SD	Control Total	Weight	Std. Mean Difference IV, Random, 95% CI
**1.1.1 Age male**								
DeRidder 2016	14.3	1.2	12	12.5	2.1	121	7.2%	0.88 [0.27, 1.48]
Engebretsen 2010	24	0.6	42	24	0.2	458	11.9%	0.00 [-0.32, 0.32]
Fousekis 2012	24.23	5.76	17	25.41	2.45	83	8.3%	-0.36 [-0.89, 0.16]
Gribble 2015	17.33	2.28	54	17.32	2.28	485	12.5%	0.00 [-0.28, 0.29]
Kawaguchi 2021	19.9	1	31	19.8	1.2	114	10.4%	0.09 [-0.31, 0.48]
Powers 2017	20.9	5.9	25	19.6	5.1	160	10.0%	0.25 [-0.17, 0.67]
Saki 2021	16.21	4.17	34	13.95	2.3	118	10.5%	0.80 [0.41, 1.19]
Willems 2005a	18.35	0.78	44	18.33	1.18	197	11.7%	0.02 [-0.31, 0.34]
**Subtotal (95% CI)**			**259**			**1736**	**82.5%**	**0.19 [-0.06, 0.43]**
Heterogeneity: Tau² = 0.08; Chi² = 22.57, df = 7 (P = 0.002); I² = 69%								
Test for overall effect: Z = 1.48 (P = 0.14)								
**1.1.2 Age female**								
Attenborough 2017	20.3	3.4	11	21.7	6.6	83	6.9%	-0.22 [-0.85, 0.41]
Willems 2005b	18.13	0.49	32	18.39	1.22	127	10.6%	-0.23 [-0.62, 0.16]
**Subtotal (95% CI)**			**43**			**210**	**17.5%**	**-0.23 [-0.56, 0.10]**
Heterogeneity: Tau² = 0.00; Chi² = 0.00, df = 1 (P = 0.97); I² = 0%								
Test for overall effect: Z = 1.36 (P = 0.18)								
**Total (95% CI)**			**302**			**1946**	**100.0%**	**0.11 [-0.11, 0.33]**
Heterogeneity: Tau² = 0.08; Chi² = 26.87, df = 9 (P = 0.001); I² = 67%								
Test for overall effect: Z = 1.01 (P = 0.31)								
Test for subgroup differences: Chi² = 3.89, df = 1 (P = 0.05), I² = 74.3%								

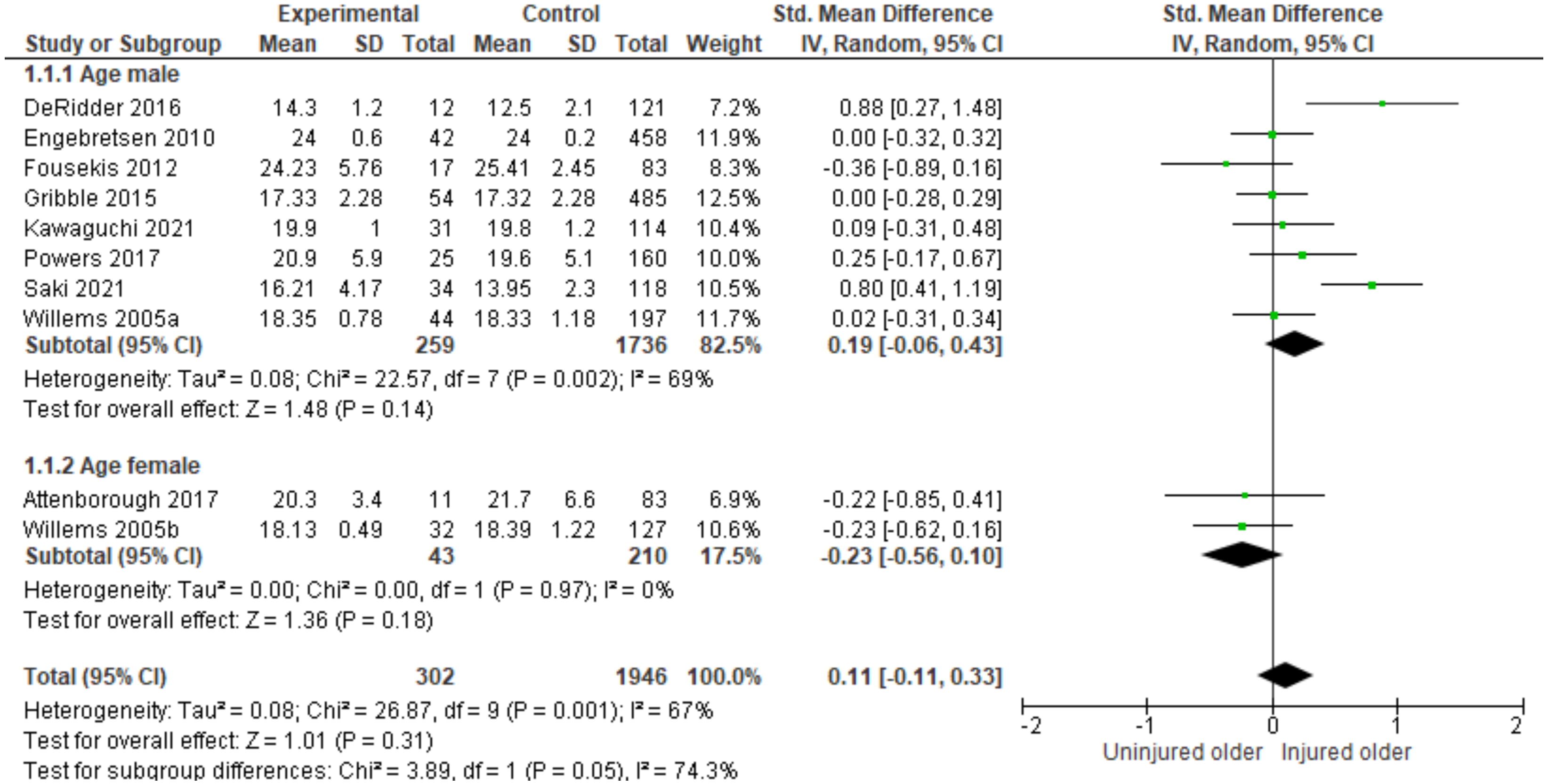

Supp Fig 1. Forest plot depicting the meta-analytical results for *age* as a risk factor for ankle sprain injury in males and females

	Injured			Uninjured				Std. Mean Difference
Study or Subgroup	Mean	SD	Total	Mean	SD	Total	Weight	IV, Random, 95% CI
**1.2.1 Weight male**								
Beynnon 2001	78	4.1	7	76.7	7.2	43	3.7%	0.19 [-0.61, 0.99]
Engebretsen 2010	77.9	0.4	43	78	1.1	450	12.0%	-0.09 [-0.41, 0.22]
Fousekis 2012	73.82	5.81	17	70.98	6.17	83	6.9%	0.46 [-0.06, 0.99]
Gribble 2015	96.15	23.92	54	87.97	20.8	485	12.9%	0.39 [0.10, 0.67]
Kawaguchi 2021	66.3	6.2	31	67.6	6.4	114	9.6%	-0.20 [-0.60, 0.19]
Powers 2017	71.6	7.1	25	68.6	10.3	160	9.0%	0.30 [-0.12, 0.72]
Saki 2021	64.06	15.74	34	53.17	16.05	118	9.8%	0.68 [0.29, 1.07]
Willems 2005a	71.14	5.94	44	69.24	7.19	197	11.5%	0.27 [-0.06, 0.60]
**Subtotal (95% CI)**			**255**			**1650**	**75.3%**	**0.24 [0.03, 0.45]**
Heterogeneity: Tau² = 0.05; Chi² = 15.87, df = 7 (P = 0.03); I² = 56%								
Test for overall effect: Z = 2.26 (P = 0.02)								
**1.2.2 Weight female**								
Attenborough 2017	71.9	14.1	11	69.8	14.5	83	5.4%	0.14 [-0.49, 0.77]
Beynnon 2001	58.1	6.4	13	60.3	6.4	55	5.6%	-0.34 [-0.95, 0.27]
McCann 2018	60.7	6.1	8	60.6	4.1	35	3.9%	0.02 [-0.75, 0.79]
Willems 2005b	60.58	7.51	32	60.06	7.11	127	9.8%	0.07 [-0.32, 0.46]
**Subtotal (95% CI)**			**64**			**300**	**24.7%**	**-0.00 [-0.27, 0.27]**
Heterogeneity: Tau² = 0.00; Chi² = 1.54, df = 3 (P = 0.67); I² = 0%								
Test for overall effect: Z = 0.02 (P = 0.98)								
**Total (95% CI)**			**319**			**1950**	**100.0%**	**0.18 [0.01, 0.35]**
Heterogeneity: Tau² = 0.04; Chi² = 19.84, df = 11 (P = 0.05); I² = 45%								
Test for overall effect: Z = 2.06 (P = 0.04)								
Test for subgroup differences: Chi² = 1.97, df = 1 (P = 0.16), I² = 49.3%								

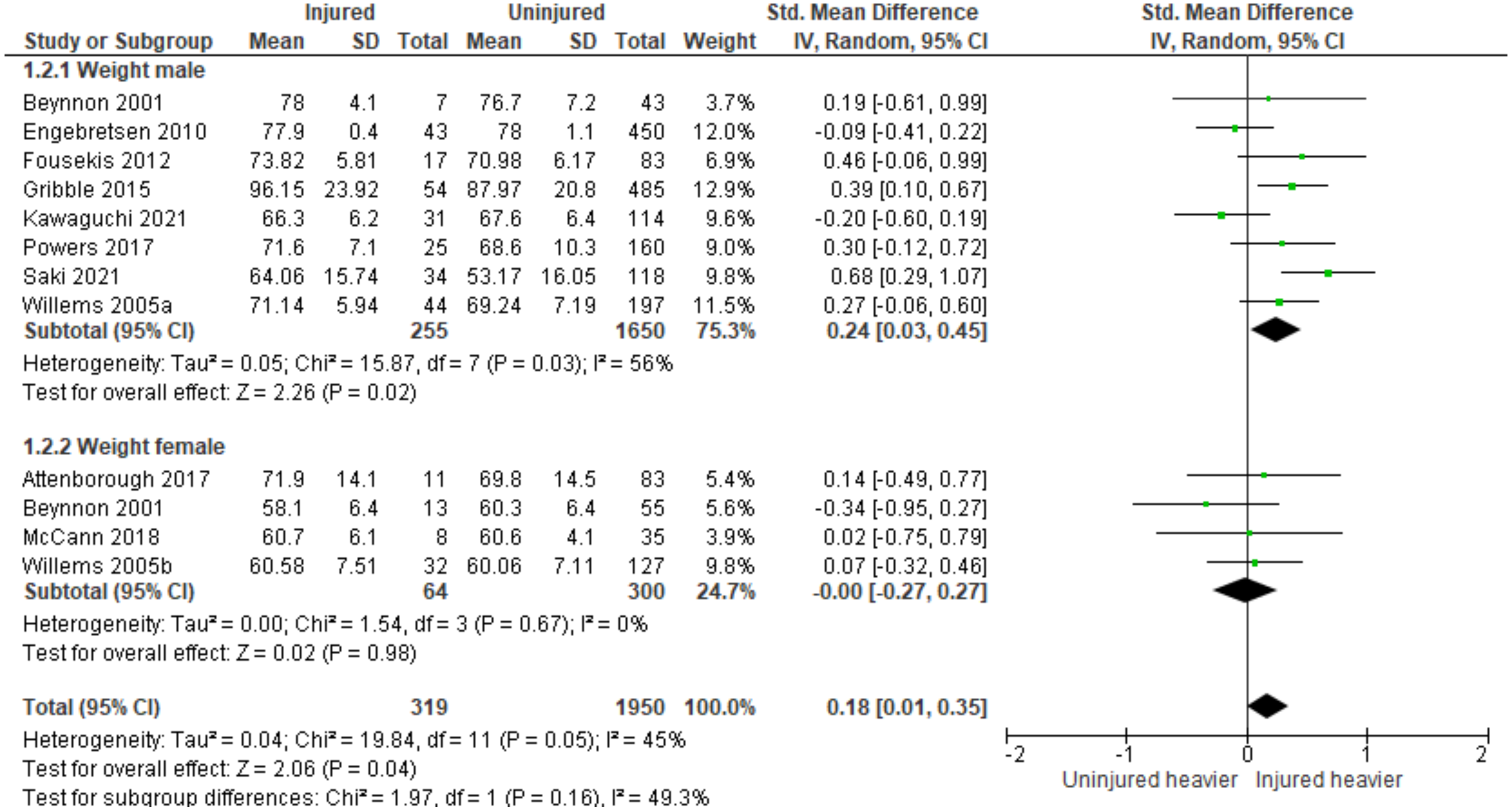

Supp Fig 2. Forest plot depicting the meta-analytical results for *weight* as a risk factor for ankle sprain injury in males and females

Study or Subgroup	Injured Mean	Injured SD	Injured Total	Uninjured Mean	Uninjured SD	Uninjured Total	Weight	Std. Mean Difference IV, Random, 95% CI
**1.3.1 Height male**								
Engebretsen 2010	181	1	42	181.4	0.3	455	11.1%	-0.98 [-1.30, -0.66]
Fousekis 2012	177.07	5.93	17	177.78	5.6	83	9.3%	-0.12 [-0.65, 0.40]
Gribble 2015	180.43	8.66	54	180.6	9.12	485	11.5%	-0.02 [-0.30, 0.26]
Kawaguchi 2021	173	5	31	173.7	6.2	114	10.5%	-0.12 [-0.51, 0.28]
Powers 2017	178	6	25	178	8	160	10.2%	0.00 [-0.42, 0.42]
Saki 2021	173.26	15.32	34	163.42	14.67	118	10.5%	0.66 [0.27, 1.05]
Willems 2005a	180.73	5.71	44	179.88	6.26	197	11.1%	0.14 [-0.19, 0.46]
**Subtotal (95% CI)**			**247**			**1612**	**74.3%**	**-0.07 [-0.45, 0.32]**
Heterogeneity: Tau² = 0.23; Chi² = 46.12, df = 6 (P < 0.00001); I² = 87%								
Test for overall effect: Z = 0.34 (P = 0.73)								
**1.3.2 Height female**								
Attenborough 2017	169.7	6	11	170.2	6.9	83	8.3%	-0.07 [-0.70, 0.56]
McCann 2018	169.2	2.3	8	166.3	3.7	35	6.9%	0.81 [0.02, 1.60]
Willems 2005b	167.01	5.95	32	167.37	5.19	127	10.6%	-0.07 [-0.45, 0.32]
**Subtotal (95% CI)**			**51**			**245**	**25.7%**	**0.14 [-0.34, 0.62]**
Heterogeneity: Tau² = 0.09; Chi² = 4.08, df = 2 (P = 0.13); I² = 51%								
Test for overall effect: Z = 0.57 (P = 0.57)								
**Total (95% CI)**			**298**			**1857**	**100.0%**	**-0.01 [-0.31, 0.30]**
Heterogeneity: Tau² = 0.19; Chi² = 51.09, df = 9 (P < 0.00001); I² = 82%								
Test for overall effect: Z = 0.05 (P = 0.96)								
Test for subgroup differences: Chi² = 0.43, df = 1 (P = 0.51), I² = 0%								

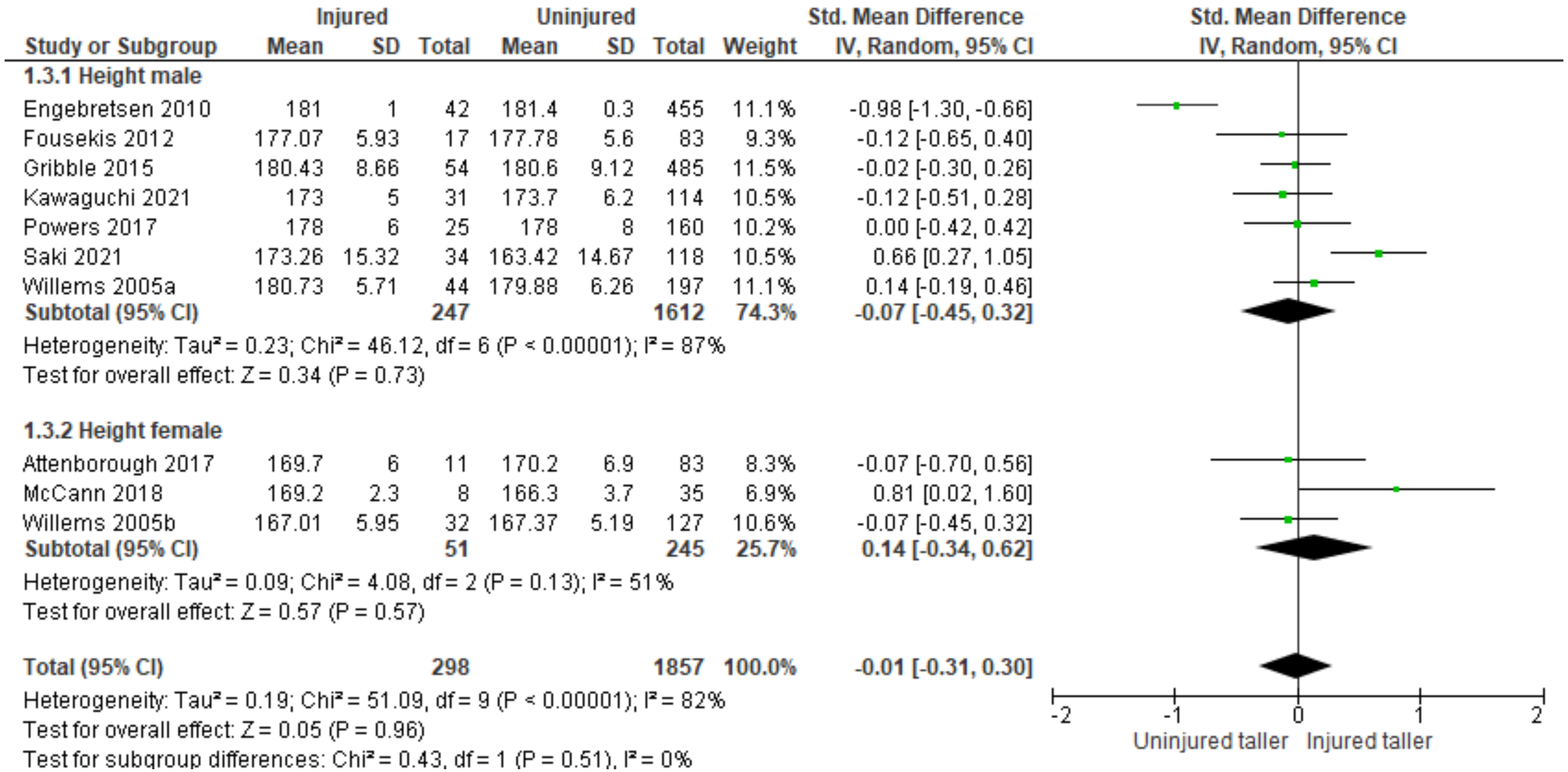

Supp Fig 3. Forest plot depicting the meta-analytical results for *height* as a risk factor for ankle sprain injury in males and females

Study or Subgroup	Injured Mean	Injured SD	Injured Total	Uninjured Mean	Uninjured SD	Uninjured Total	Weight	Std. Mean Difference IV, Random, 95% CI
**1.4.1 BMI male**								
DeRidder 2016	19.8	1.9	12	17.6	1.9	121	6.5%	1.15 [0.54, 1.76]
Engebretsen 2010	23.8	0.2	42	23.7	0.1	444	9.5%	0.89 [0.57, 1.21]
Fousekis 2012	23.44	1.42	17	22.6	0.94	83	7.2%	0.81 [0.27, 1.34]
Gribble 2015	29.32	6.08	54	26.7	4.64	485	9.9%	0.54 [0.26, 0.83]
Hartley 2018	28.87	5.09	39	26.78	5.88	342	9.4%	0.36 [0.03, 0.69]
Kawaguchi 2021	22.1	1.7	31	22.4	1.4	114	8.7%	-0.20 [-0.60, 0.19]
Powers 2017	22.5	1.1	25	21.6	2	160	8.4%	0.47 [0.05, 0.89]
Saki 2021	21.2	3.76	34	19.62	3.47	118	8.8%	0.44 [0.06, 0.83]
Willems 2005a	21.81	1.71	44	21.37	1.66	197	9.5%	0.26 [-0.06, 0.59]
**Subtotal (95% CI)**			**298**			**2064**	**78.0%**	**0.50 [0.27, 0.73]**
Heterogeneity: Tau² = 0.08; Chi² = 26.16, df = 8 (P = 0.0010); I² = 69%								
Test for overall effect: Z = 4.24 (P < 0.0001)								
**1.4.2 BMI female**								
Hartley 2018	23.12	2.32	21	24.27	3.56	145	8.0%	-0.33 [-0.79, 0.13]
McCann 2018	21.2	2.2	8	22	1.5	35	5.1%	-0.48 [-1.25, 0.30]
Willems 2005b	21.81	2.53	32	21.5	2.32	127	8.8%	0.13 [-0.26, 0.52]
**Subtotal (95% CI)**			**61**			**307**	**22.0%**	**-0.15 [-0.53, 0.22]**
Heterogeneity: Tau² = 0.04; Chi² = 3.25, df = 2 (P = 0.20); I² = 39%								
Test for overall effect: Z = 0.81 (P = 0.42)								
**Total (95% CI)**			**359**			**2371**	**100.0%**	**0.35 [0.12, 0.59]**
Heterogeneity: Tau² = 0.12; Chi² = 44.26, df = 11 (P < 0.00001); I² = 75%								
Test for overall effect: Z = 2.95 (P = 0.003)								
Test for subgroup differences: Chi² = 8.48, df = 1 (P = 0.004), I² = 88.2%								

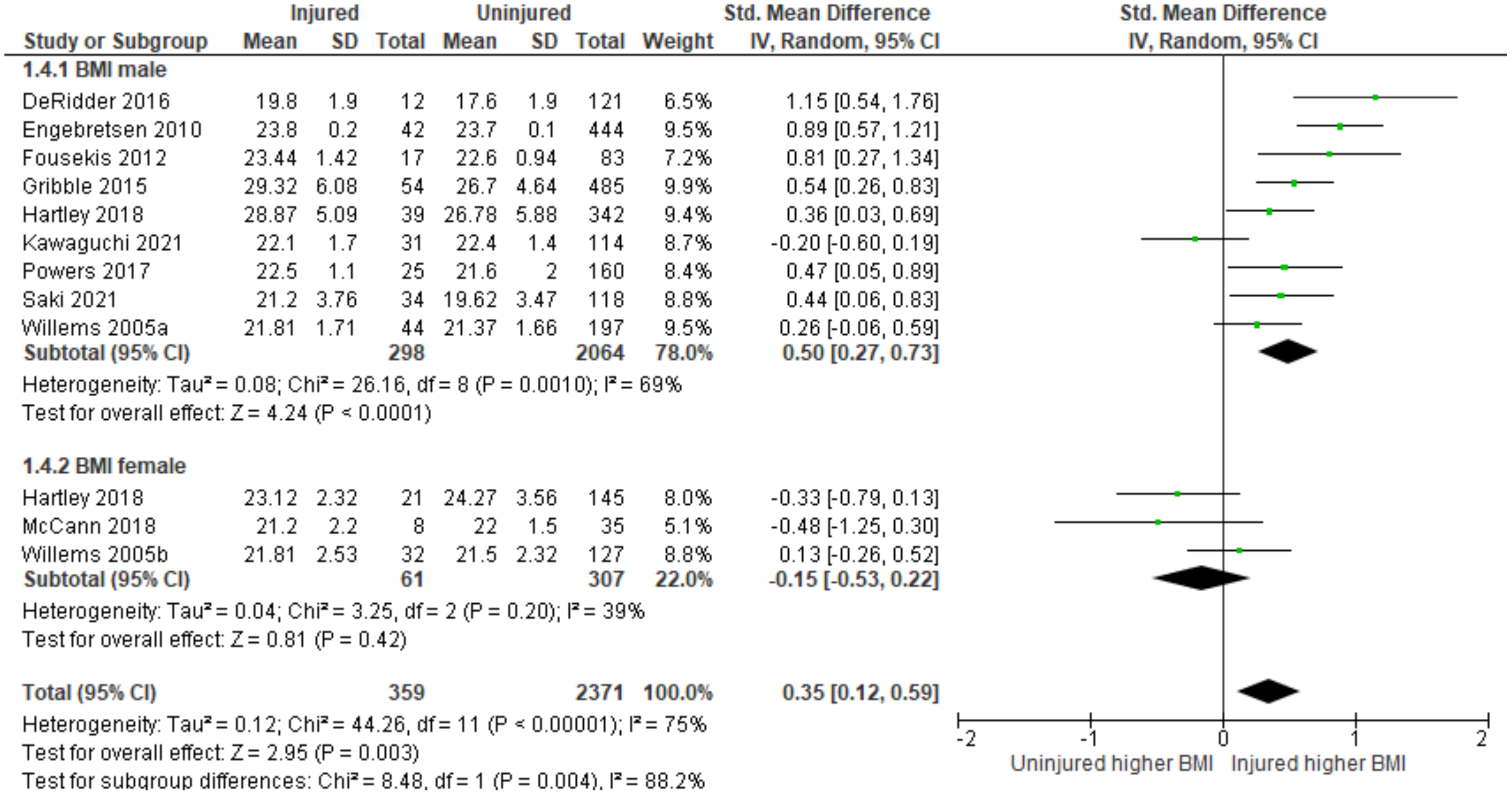

Supp Fig 4. Forest plot depicting the meta-analytical results for *body mass index* as a risk factor for ankle sprain injury in males and females

	with previous injury		without previous injury			Odds Ratio
Study or Subgroup	Events	Total	Events	Total	Weight	M-H, Random, 95% CI
**2.1.1 previous injury male**						
Engebretsen 2010	34	616	12	399	9.8%	1.88 [0.96, 3.68]
Gribble 2015	17	112	31	346	10.0%	1.82 [0.96, 3.43]
Hartley 2018	19	175	20	206	9.8%	1.13 [0.58, 2.20]
Kawaguchi 2021	15	55	16	90	9.0%	1.73 [0.78, 3.87]
Kofotolis 2007a	84	132	55	180	10.9%	3.98 [2.47, 6.40]
Powers 2017	8	36	17	149	8.2%	2.22 [0.87, 5.65]
Saki 2021	16	23	18	129	7.7%	14.10 [5.09, 39.02]
Tyler 2006	11	50	4	102	6.8%	6.91 [2.08, 23.01]
**Subtotal (95% CI)**		**1199**		**1601**	**72.2%**	**2.74 [1.66, 4.53]**
Total events	204		173			
Heterogeneity: Tau² = 0.36; Chi² = 25.41, df = 7 (P = 0.0006); I² = 72%						
Test for overall effect: Z = 3.92 (P < 0.0001)						
**2.1.2 previous injury female**						
Attenborough 2017	5	54	6	40	6.4%	0.58 [0.16, 2.05]
Hartley 2018	8	80	13	86	8.2%	0.62 [0.24, 1.60]
Kofotolis 2007b	24	138	8	64	8.6%	1.47 [0.62, 3.49]
McCann 2018	6	16	2	27	4.5%	7.50 [1.29, 43.61]
**Subtotal (95% CI)**		**288**		**217**	**27.8%**	**1.20 [0.49, 2.94]**
Total events	43		29			
Heterogeneity: Tau² = 0.48; Chi² = 7.42, df = 3 (P = 0.06); I² = 60%						
Test for overall effect: Z = 0.40 (P = 0.69)						
**Total (95% CI)**		**1487**		**1818**	**100.0%**	**2.19 [1.37, 3.49]**
Total events	247		202			
Heterogeneity: Tau² = 0.46; Chi² = 40.86, df = 11 (P < 0.0001); I² = 73%						
Test for overall effect: Z = 3.30 (P = 0.0010)						
Test for subgroup differences: Chi² = 2.48, df = 1 (P = 0.12), I² = 59.6%						

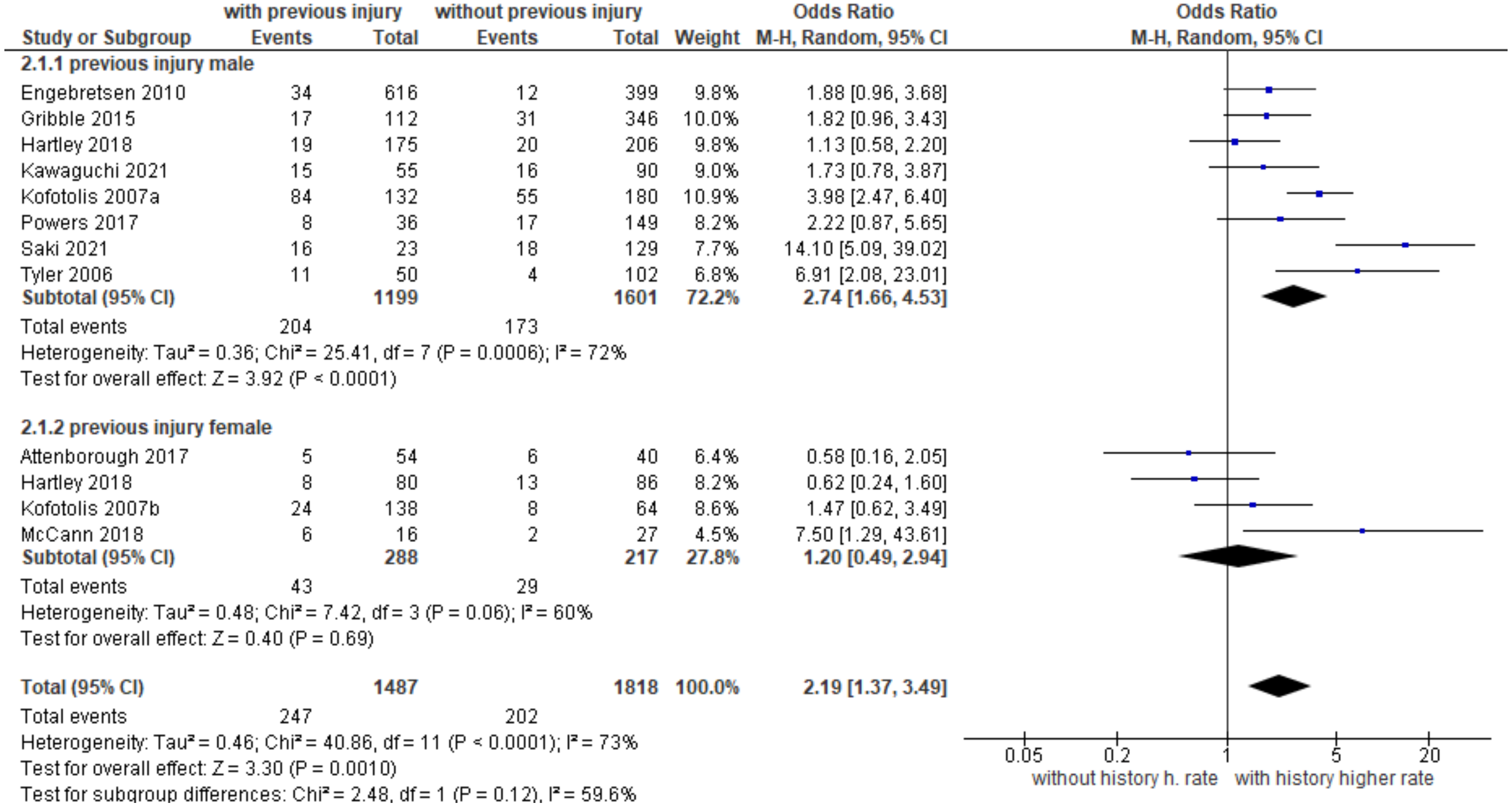

Supp Fig 5. Forest plot depicting the meta-analytical results for *previous ankle injury* as a risk factor for ankle sprain injury in males and females

Study or Subgroup	Injured Mean	Injured SD	Injured Total	Uninjured Mean	Uninjured SD	Uninjured Total	Weight	Std. Mean Difference IV, Random, 95% CI
**3.1.1 con strength plantar male**								
Beynnon 2001	77.3	13	7	71.8	13.7	43	9.6%	0.40 [-0.41, 1.20]
Willems 2005a	1.32	0.36	44	1.47	0.38	197	41.5%	-0.40 [-0.73, -0.07]
**Subtotal (95% CI)**			**51**			**240**	**51.1%**	**-0.09 [-0.85, 0.67]**
Heterogeneity: Tau² = 0.22; Chi² = 3.22, df = 1 (P = 0.07); I² = 69%								
Test for overall effect: Z = 0.23 (P = 0.82)								
**3.1.2 con strength plantar female**								
Beynnon 2001	52.3	12.3	13	53.1	11.3	55	16.0%	-0.07 [-0.67, 0.54]
Willems 2005b	1.32	0.36	32	1.38	0.38	127	32.9%	-0.16 [-0.55, 0.23]
**Subtotal (95% CI)**			**45**			**182**	**48.9%**	**-0.13 [-0.46, 0.19]**
Heterogeneity: Tau² = 0.00; Chi² = 0.06, df = 1 (P = 0.81); I² = 0%								
Test for overall effect: Z = 0.80 (P = 0.43)								
**Total (95% CI)**			**96**			**422**	**100.0%**	**-0.19 [-0.45, 0.07]**
Heterogeneity: Tau² = 0.01; Chi² = 3.72, df = 3 (P = 0.29); I² = 19%								
Test for overall effect: Z = 1.43 (P = 0.15)								
Test for subgroup differences: Chi² = 0.01, df = 1 (P = 0.92), I² = 0%								

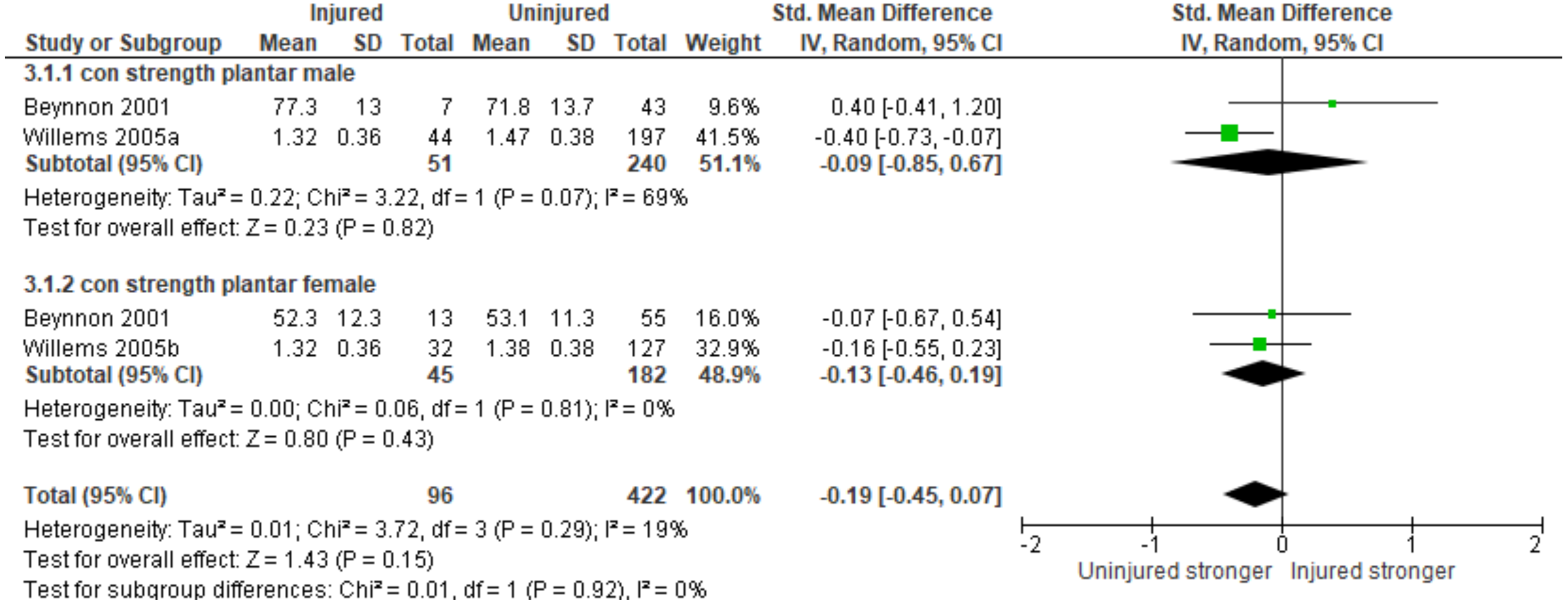

Supp Fig 6. Forest plot depicting the meta-analytical results for *concentric plantarflexion strength* as a risk factor for ankle sprain injury in males and females

Study or Subgroup	Injured Mean	Injured SD	Injured Total	Uninjured Mean	Uninjured SD	Uninjured Total	Weight	Std. Mean Difference IV, Random, 95% CI
**3.2.1 con strength dorsi male**								
Beynnon 2001	28.1	4.2	7	26.8	3.8	43	12.4%	0.33 [-0.47, 1.13]
Willems 2005a	0.54	0.21	44	0.73	0.3	197	37.0%	-0.66 [-0.99, -0.33]
**Subtotal (95% CI)**			**51**			**240**	**49.4%**	**-0.23 [-1.20, 0.73]**
Heterogeneity: Tau² = 0.40; Chi² = 5.05, df = 1 (P = 0.02); I² = 80%								
Test for overall effect: Z = 0.48 (P = 0.63)								
**3.2.2 con strength dorsi female**								
Beynnon 2001	17	2.4	13	18.2	2.6	54	18.7%	-0.46 [-1.07, 0.15]
Willems 2005b	0.56	0.31	32	0.71	0.31	127	31.9%	-0.48 [-0.87, -0.09]
**Subtotal (95% CI)**			**45**			**181**	**50.6%**	**-0.48 [-0.81, -0.15]**
Heterogeneity: Tau² = 0.00; Chi² = 0.00, df = 1 (P = 0.96); I² = 0%								
Test for overall effect: Z = 2.83 (P = 0.005)								
**Total (95% CI)**			**96**			**421**	**100.0%**	**-0.44 [-0.76, -0.13]**
Heterogeneity: Tau² = 0.04; Chi² = 5.08, df = 3 (P = 0.17); I² = 41%								
Test for overall effect: Z = 2.75 (P = 0.006)								
Test for subgroup differences: Chi² = 0.21, df = 1 (P = 0.64), I² = 0%								

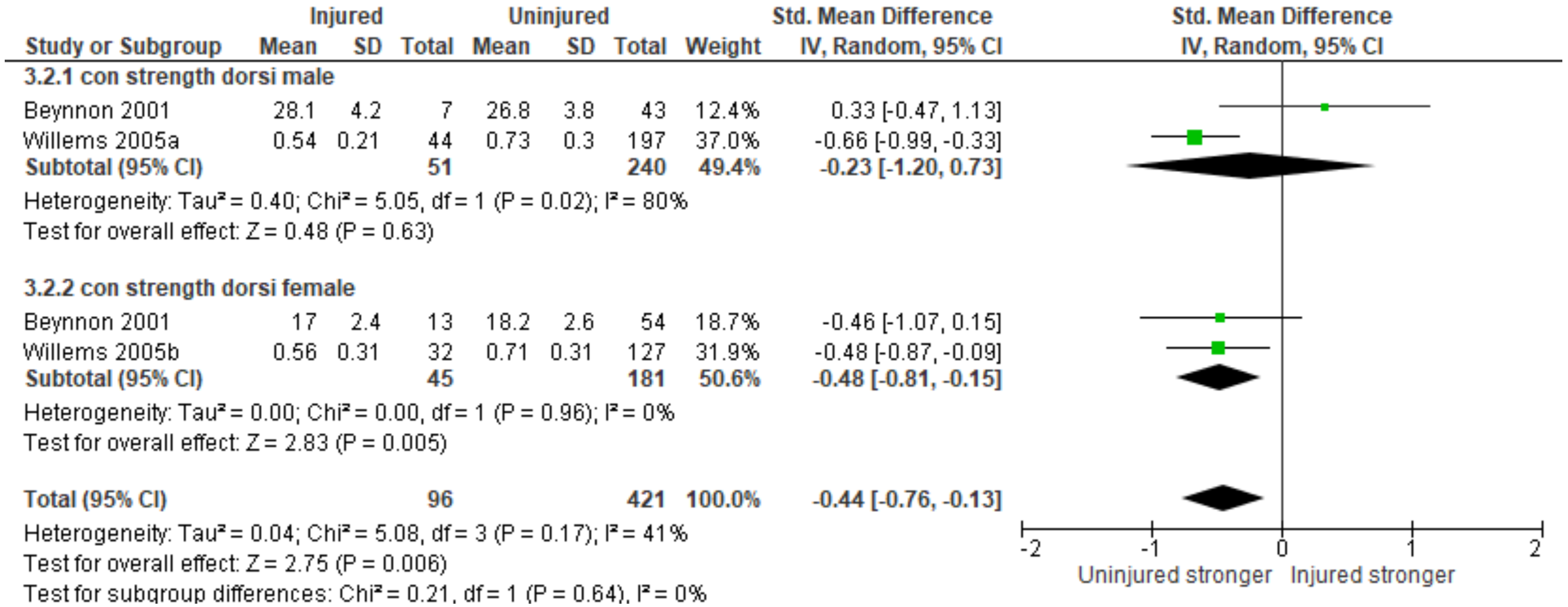

Supp Fig 7. Forest plot depicting the meta-analytical results for *concentric dorsiflexion strength* as a risk factor for ankle sprain injury in males and females

Study or Subgroup	Injured Mean	Injured SD	Injured Total	Uninjured Mean	Uninjured SD	Uninjured Total	Weight	Std. Mean Difference IV, Random, 95% CI
**3.3.1 con strength inversion male**								
Beynnon 2001	21.1	5.1	7	20	5.3	43	7.7%	0.21 [-0.59, 1.01]
Willems 2005a	0.51	0.12	44	0.54	0.16	197	46.1%	-0.19 [-0.52, 0.13]
**Subtotal (95% CI)**			**51**			**240**	**53.8%**	**-0.14 [-0.44, 0.17]**
Heterogeneity: Tau² = 0.00; Chi² = 0.82, df = 1 (P = 0.36); I² = 0%								
Test for overall effect: Z = 0.89 (P = 0.37)								
**3.3.2 con strength inversion female**								
Beynnon 2001	15.2	5.9	13	14.2	4.2	54	13.4%	0.22 [-0.39, 0.82]
Willems 2005b	0.5	0.22	32	0.53	0.22	127	32.8%	-0.14 [-0.52, 0.25]
**Subtotal (95% CI)**			**45**			**181**	**46.2%**	**-0.03 [-0.36, 0.29]**
Heterogeneity: Tau² = 0.00; Chi² = 0.92, df = 1 (P = 0.34); I² = 0%								
Test for overall effect: Z = 0.20 (P = 0.84)								
**Total (95% CI)**			**96**			**421**	**100.0%**	**-0.09 [-0.31, 0.13]**
Heterogeneity: Tau² = 0.00; Chi² = 1.95, df = 3 (P = 0.58); I² = 0%								
Test for overall effect: Z = 0.79 (P = 0.43)								
Test for subgroup differences: Chi² = 0.21, df = 1 (P = 0.65), I² = 0%								

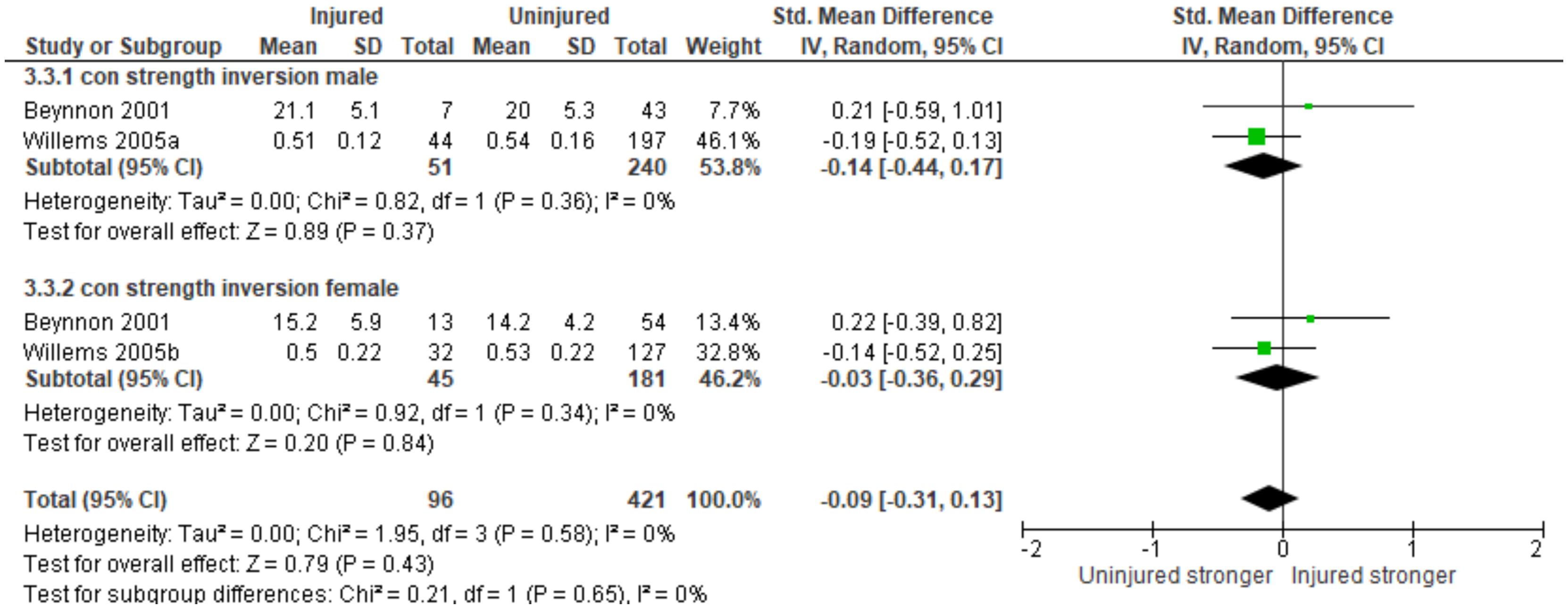

Supp Fig 8. Forest plot depicting the meta-analytical results for *concentric inversion strength* as a risk factor for ankle sprain injury in males and females

Study or Subgroup	Injured Mean	Injured SD	Injured Total	Uninjured Mean	Uninjured SD	Uninjured Total	Weight	Std. Mean Difference IV, Random, 95% CI
**3.4.1 con strength eversion male**								
Beynnon 2001	22.3	5.6	7	19.5	5.3	43	10.5%	0.52 [-0.29, 1.32]
Willems 2005a	0.45	0.14	44	0.43	0.12	197	39.9%	0.16 [-0.17, 0.49]
**Subtotal (95% CI)**			**51**			**240**	**50.4%**	**0.21 [-0.09, 0.51]**
Heterogeneity: Tau² = 0.00; Chi² = 0.64, df = 1 (P = 0.42); I² = 0%								
Test for overall effect: Z = 1.37 (P = 0.17)								
**3.4.2 con strength eversion female**								
Beynnon 2001	12.6	3.4	13	13.3	2.7	54	17.0%	-0.24 [-0.85, 0.36]
Willems 2005b	0.36	0.11	32	0.37	0.007	127	32.6%	-0.20 [-0.59, 0.19]
**Subtotal (95% CI)**			**45**			**181**	**49.6%**	**-0.21 [-0.54, 0.11]**
Heterogeneity: Tau² = 0.00; Chi² = 0.01, df = 1 (P = 0.91); I² = 0%								
Test for overall effect: Z = 1.28 (P = 0.20)								
**Total (95% CI)**			**96**			**421**	**100.0%**	**0.01 [-0.27, 0.29]**
Heterogeneity: Tau² = 0.02; Chi² = 4.15, df = 3 (P = 0.25); I² = 28%								
Test for overall effect: Z = 0.08 (P = 0.94)								
Test for subgroup differences: Chi² = 3.49, df = 1 (P = 0.06), I² = 71.4%								

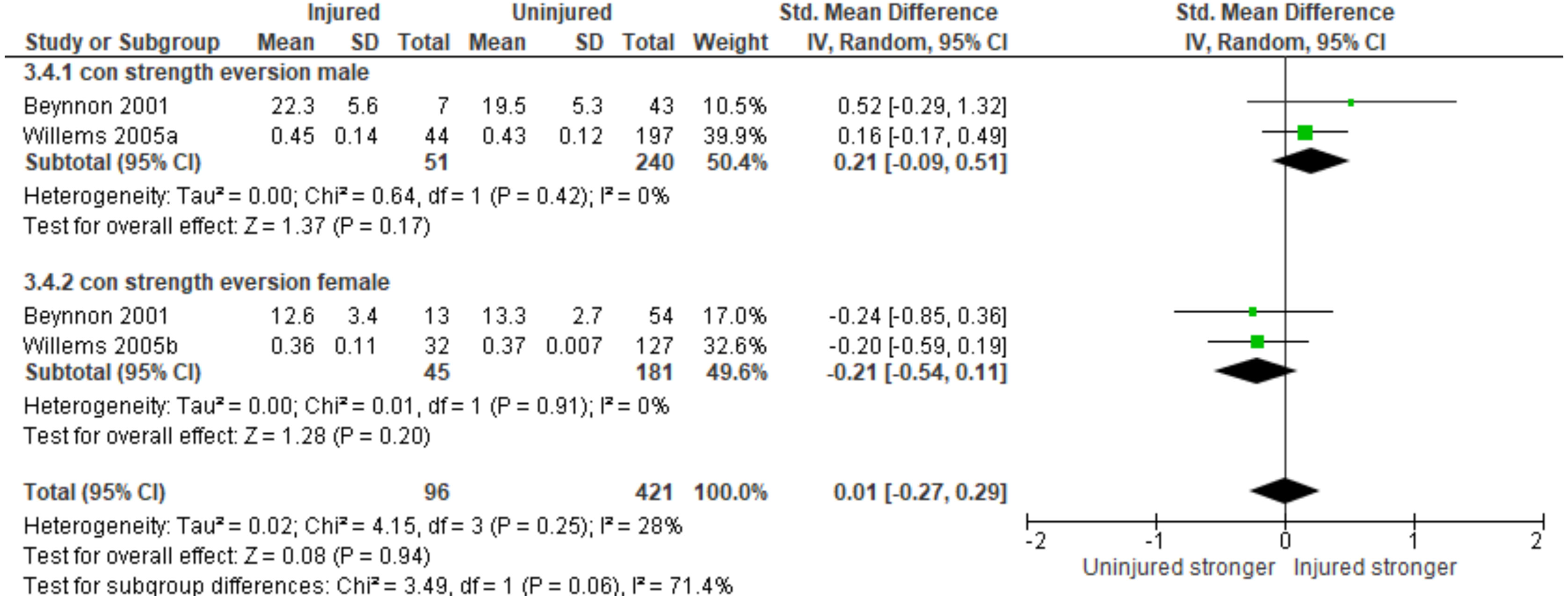

Supp Fig 9. Forest plot depicting the meta-analytical results for *concentric eversion strength* as a risk factor for ankle sprain injury in males and females

Study or Subgroup	Injured Mean	Injured SD	Injured Total	Uninjured Mean	Uninjured SD	Uninjured Total	Weight	Std. Mean Difference IV, Random, 95% CI
**4.1.1 ecc strength inversion male**								
Beynnon 2001	25.7	6.2	7	23.8	6.2	43	8.4%	0.30 [-0.50, 1.10]
Willems 2005a	0.53	0.13	44	0.56	0.18	197	44.4%	-0.17 [-0.50, 0.15]
**Subtotal (95% CI)**			**51**			**240**	**52.8%**	**-0.08 [-0.45, 0.28]**
Heterogeneity: Tau² = 0.02; Chi² = 1.16, df = 1 (P = 0.28); I² = 14%								
Test for overall effect: Z = 0.44 (P = 0.66)								
**4.1.2 ecc strength inversion female**								
Beynnon 2001	17.3	3.7	13	16.3	4.3	54	14.4%	0.24 [-0.37, 0.84]
Willems 2005b	0.49	0.18	32	0.55	0.21	127	32.8%	-0.29 [-0.68, 0.10]
**Subtotal (95% CI)**			**45**			**181**	**47.2%**	**-0.08 [-0.59, 0.42]**
Heterogeneity: Tau² = 0.07; Chi² = 2.06, df = 1 (P = 0.15); I² = 51%								
Test for overall effect: Z = 0.32 (P = 0.75)								
**Total (95% CI)**			**96**			**421**	**100.0%**	**-0.11 [-0.35, 0.12]**
Heterogeneity: Tau² = 0.00; Chi² = 3.24, df = 3 (P = 0.36); I² = 7%								
Test for overall effect: Z = 0.95 (P = 0.34)								
Test for subgroup differences: Chi² = 0.00, df = 1 (P = 1.00), I² = 0%								

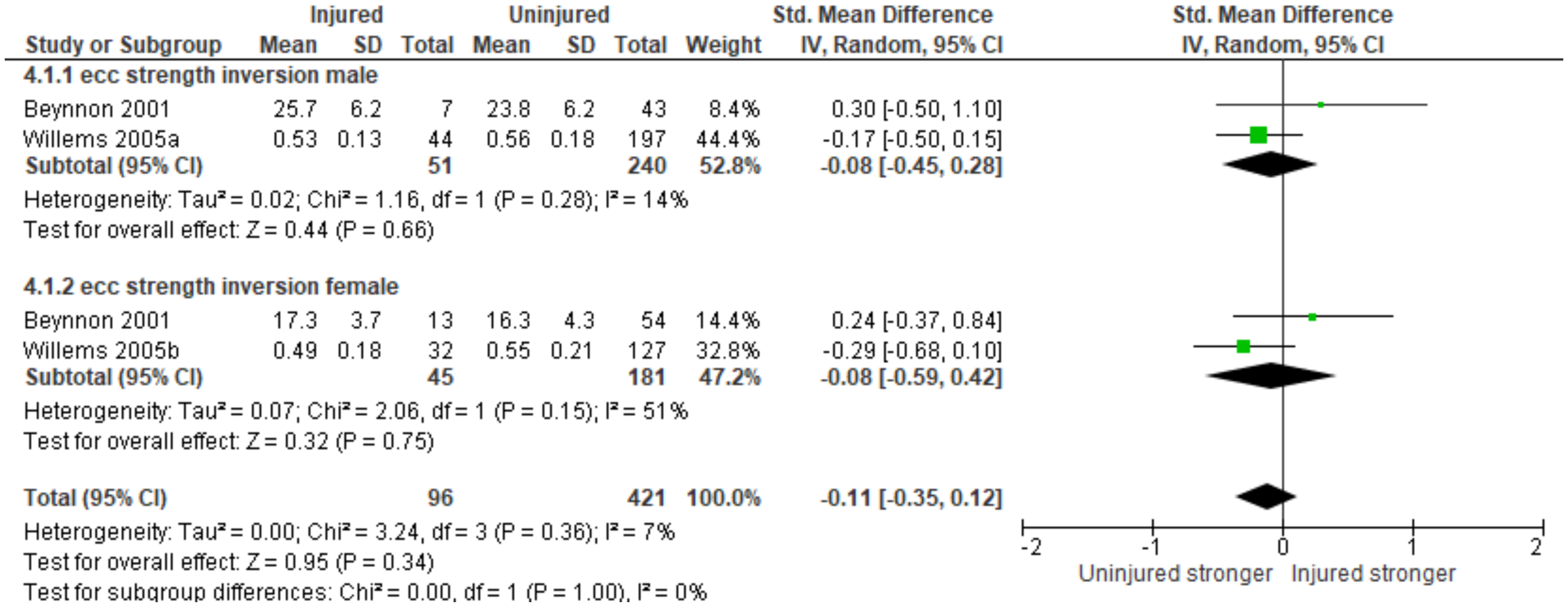

Supp Fig 10. Forest plot depicting the meta-analytical results for *eccentric inversion strength* as a risk factor for ankle sprain injury in males and females

Study or Subgroup	Injured Mean	Injured SD	Injured Total	Uninjured Mean	Uninjured SD	Uninjured Total	Weight	Std. Mean Difference IV, Random, 95% CI
**4.2.1 ecc strength eversion male**								
Beynnon 2001	24	5.4	7	24.6	5.8	43	7.7%	-0.10 [-0.90, 0.70]
Willems 2005a	0.48	0.14	44	0.46	0.1	197	46.1%	0.18 [-0.14, 0.51]
**Subtotal (95% CI)**			**51**			**240**	**53.8%**	**0.14 [-0.16, 0.45]**
Heterogeneity: Tau² = 0.00; Chi² = 0.42, df = 1 (P = 0.52); I² = 0%								
Test for overall effect: Z = 0.93 (P = 0.35)								
**4.2.2 ecc strength eversion female**								
Beynnon 2001	15.5	3.3	13	16.2	3.8	54	13.4%	-0.19 [-0.79, 0.42]
Willems 2005b	0.41	0.1	32	0.42	0.11	127	32.8%	-0.09 [-0.48, 0.30]
**Subtotal (95% CI)**			**45**			**181**	**46.2%**	**-0.12 [-0.45, 0.21]**
Heterogeneity: Tau² = 0.00; Chi² = 0.07, df = 1 (P = 0.80); I² = 0%								
Test for overall effect: Z = 0.72 (P = 0.47)								
**Total (95% CI)**			**96**			**421**	**100.0%**	**0.02 [-0.20, 0.24]**
Heterogeneity: Tau² = 0.00; Chi² = 1.82, df = 3 (P = 0.61); I² = 0%								
Test for overall effect: Z = 0.19 (P = 0.85)								
Test for subgroup differences: Chi² = 1.33, df = 1 (P = 0.25), I² = 25.0%								

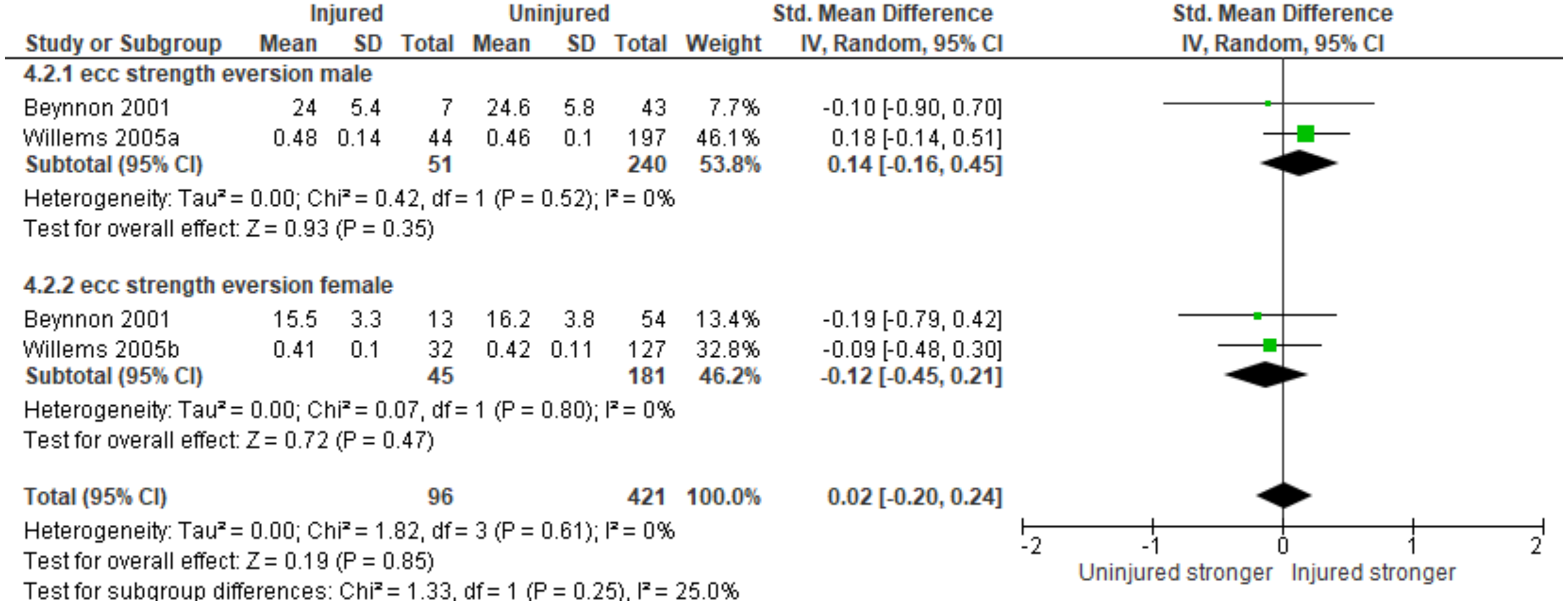

Supp Fig 11. Forest plot depicting the meta-analytical results for *eccentric eversion strength* as a risk factor for ankle sprain injury in males and females

	Injured			Uninjured				Std. Mean Difference
Study or Subgroup	Mean	SD	Total	Mean	SD	Total	Weight	IV, Random, 95% CI
**5.1.1 isometric hip abduction male**								
DeRidder 2016	1.2	0.5	12	1.4	0.4	121	18.3%	-0.49 [-1.08, 0.11]
Kawaguchi 2021	2.3	0.5	31	2.5	0.4	228	45.7%	-0.48 [-0.86, -0.11]
Powers 2017	35.7	5.6	25	39.5	6.6	160	35.9%	-0.58 [-1.01, -0.16]
**Subtotal (95% CI)**			**68**			**509**	**100.0%**	**-0.52 [-0.78, -0.26]**
Heterogeneity: Tau² = 0.00; Chi² = 0.14, df = 2 (P = 0.93); I² = 0%								
Test for overall effect: Z = 3.99 (P < 0.0001)								
**Total (95% CI)**			**68**			**509**	**100.0%**	**-0.52 [-0.78, -0.26]**
Heterogeneity: Tau² = 0.00; Chi² = 0.14, df = 2 (P = 0.93); I² = 0%								
Test for overall effect: Z = 3.99 (P < 0.0001)								
Test for subgroup differences: Not applicable								

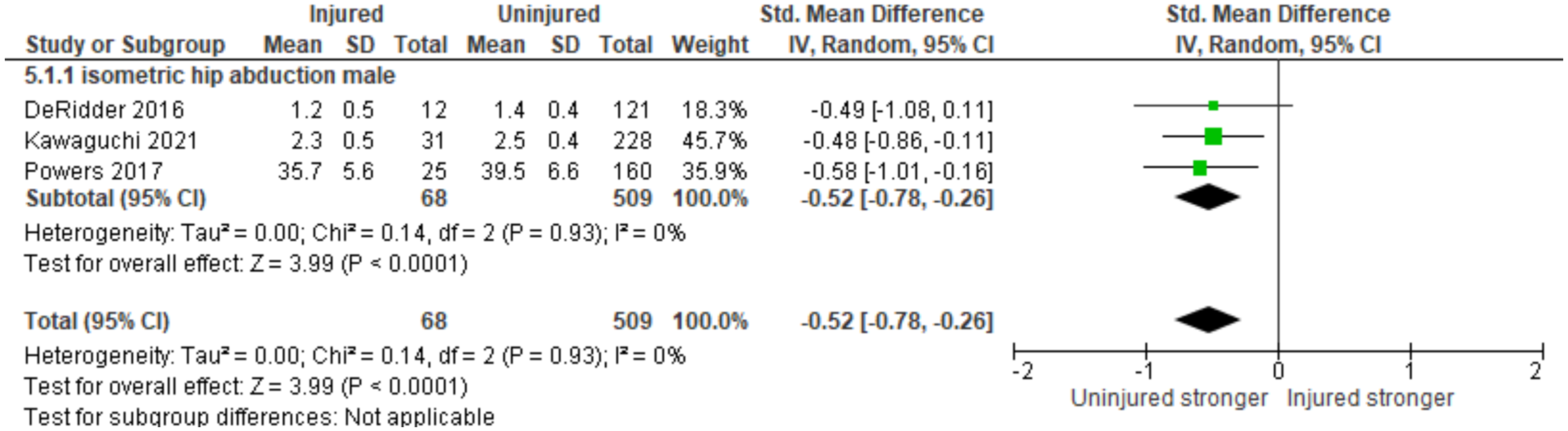

Supp Fig 12. Forest plot depicting the meta-analytical results for *isometric hip abduction strength* as a risk factor for ankle sprain injury in males.

Study or Subgroup	pathologic Events	pathologic Total	normal Events	normal Total	Weight	Odds Ratio M-H, Random, 95% CI
**6.1.1 Anterior drawer male**						
Beynnon 2001	2	11	5	37	16.4%	1.42 [0.24, 8.59]
Engebretsen 2010	9	138	29	738	39.8%	1.71 [0.79, 3.69]
Fousekis 2012	7	38	10	62	30.9%	1.17 [0.41, 3.40]
**Subtotal (95% CI)**		**187**		**837**	**87.1%**	**1.49 [0.83, 2.69]**
Total events	18		44			
Heterogeneity: Tau² = 0.00; Chi² = 0.31, df = 2 (P = 0.85); I² = 0%						
Test for overall effect: Z = 1.33 (P = 0.18)						
**6.1.2 anterior drawer female**						
Beynnon 2001	1	22	12	45	12.9%	0.13 [0.02, 1.08]
**Subtotal (95% CI)**		**22**		**45**	**12.9%**	**0.13 [0.02, 1.08]**
Total events	1		12			
Heterogeneity: Not applicable						
Test for overall effect: Z = 1.89 (P = 0.06)						
**Total (95% CI)**		**209**		**882**	**100.0%**	**1.06 [0.45, 2.50]**
Total events	19		56			
Heterogeneity: Tau² = 0.33; Chi² = 5.38, df = 3 (P = 0.15); I² = 44%						
Test for overall effect: Z = 0.13 (P = 0.90)						
Test for subgroup differences: Chi² = 4.73, df = 1 (P = 0.03), I² = 78.8%						

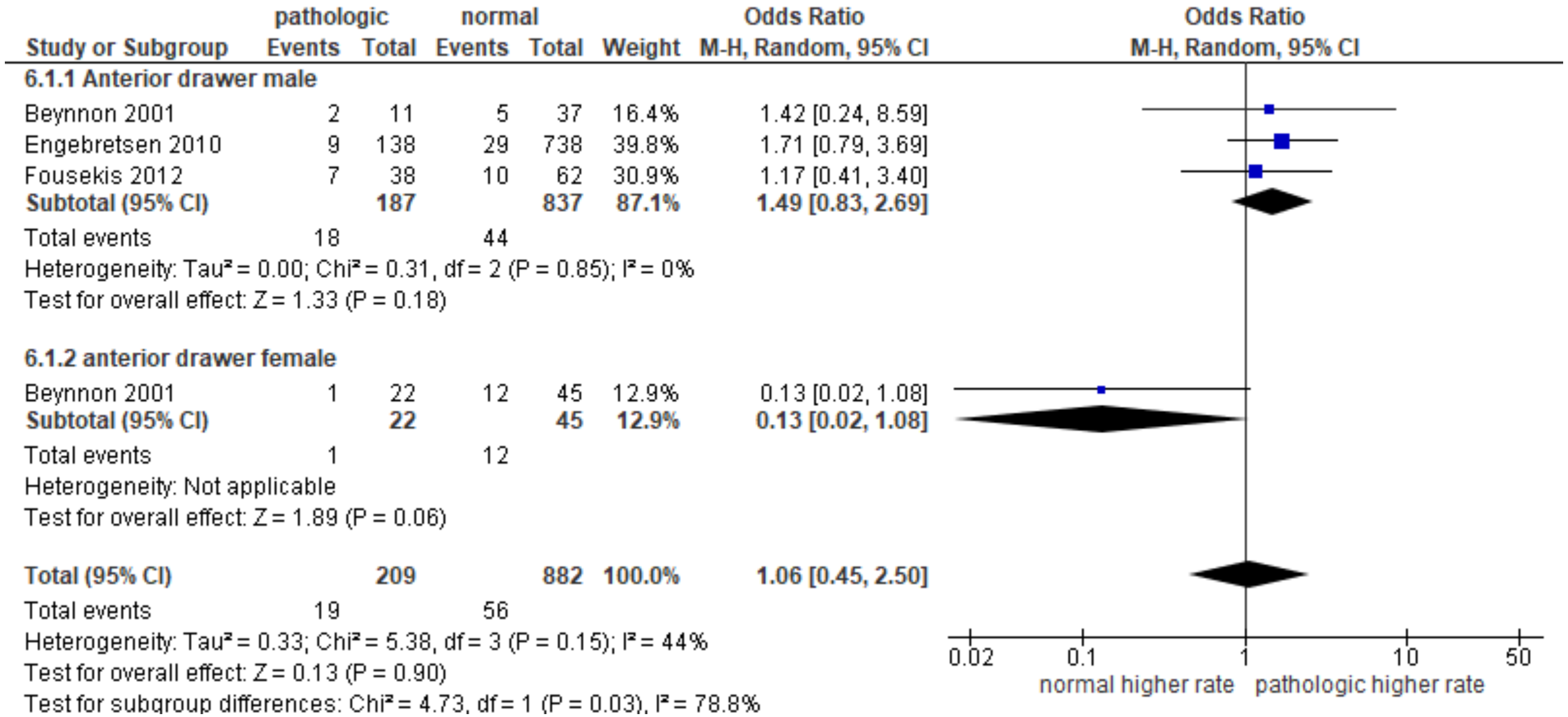

Supp Fig 13. Forest plot depicting the meta-analytical results for *ankle anterior drawer test (joint laxity)* as a risk factor for ankle sprain injury in males and females

Study or Subgroup	Injured Mean	Injured SD	Injured Total	Uninjured Mean	Uninjured SD	Uninjured Total	Weight	Std. Mean Difference IV, Random, 95% CI
**7.1.1 anterior male**								
Gribble 2015	65.57	7.9	54	69.72	7.53	485	42.9%	-0.55 [-0.83, -0.26]
Hartley 2018	55.61	6.7	39	58.24	6.78	342	31.4%	-0.39 [-0.72, -0.06]
**Subtotal (95% CI)**			**93**			**827**	**74.3%**	**-0.48 [-0.70, -0.26]**
Heterogeneity: Tau² = 0.00; Chi² = 0.52, df = 1 (P = 0.47); I² = 0%								
Test for overall effect: Z = 4.37 (P < 0.0001)								
**7.1.2 anterior female**								
Attenborough 2017	65.6	6.2	11	66.3	5.1	83	8.9%	-0.13 [-0.76, 0.50]
Hartley 2018	62.02	7.71	21	62.86	6.12	145	16.7%	-0.13 [-0.59, 0.33]
**Subtotal (95% CI)**			**32**			**228**	**25.7%**	**-0.13 [-0.50, 0.24]**
Heterogeneity: Tau² = 0.00; Chi² = 0.00, df = 1 (P = 1.00); I² = 0%								
Test for overall effect: Z = 0.70 (P = 0.48)								
**Total (95% CI)**			**125**			**1055**	**100.0%**	**-0.39 [-0.58, -0.20]**
Heterogeneity: Tau² = 0.00; Chi² = 3.05, df = 3 (P = 0.38); I² = 2%								
Test for overall effect: Z = 4.06 (P < 0.0001)								
Test for subgroup differences: Chi² = 2.54, df = 1 (P = 0.11), I² = 60.6%								

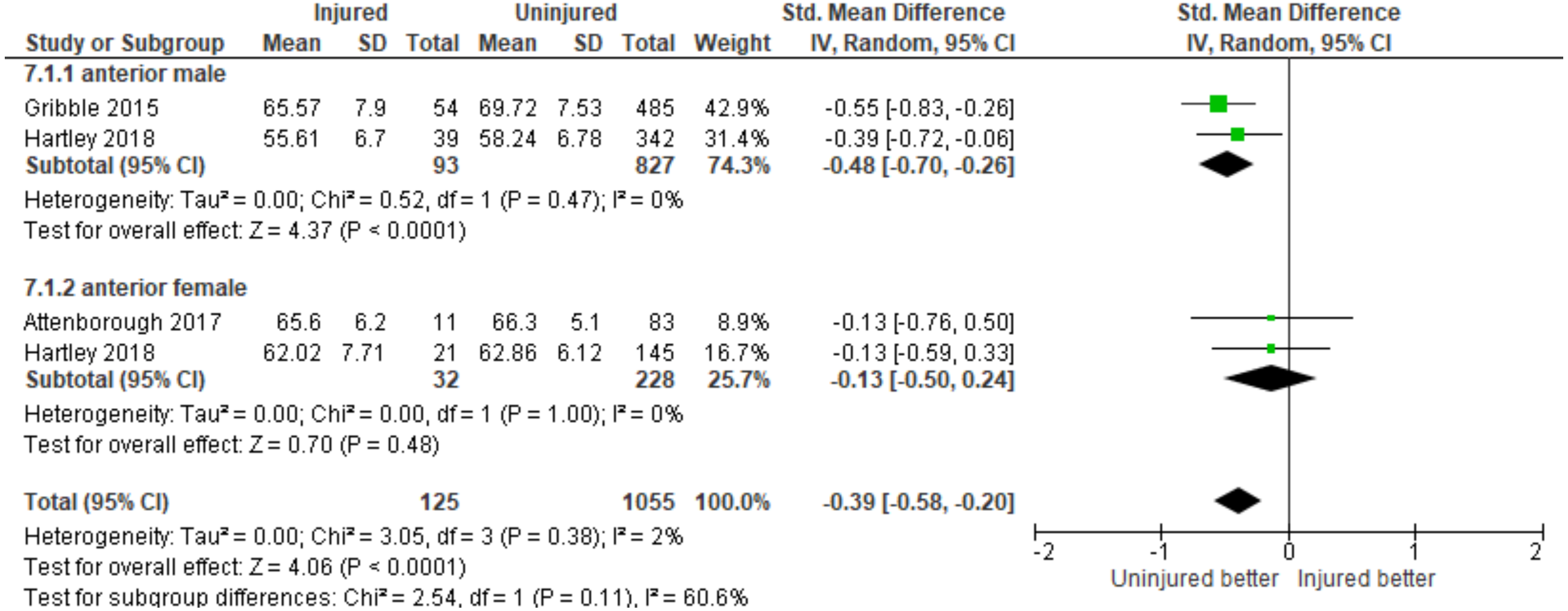

Supp Fig 14. Forest plot depicting the meta-analytical results for *anterior dynamic balance* as a risk factor for ankle sprain injury in males and females

Study or Subgroup	Injured Mean	Injured SD	Injured Total	Uninjured Mean	Uninjured SD	Uninjured Total	Weight	Std. Mean Difference IV, Random, 95% CI
**7.2.1 post-lat male**								
Gribble 2015	71.29	11.04	54	74.6	11.38	485	32.2%	-0.29 [-0.57, -0.01]
Hartley 2018	99.21	9.26	39	100.67	11.33	342	29.3%	-0.13 [-0.46, 0.20]
**Subtotal (95% CI)**			**93**			**827**	**61.5%**	**-0.22 [-0.44, -0.01]**
Heterogeneity: Tau² = 0.00; Chi² = 0.52, df = 1 (P = 0.47); I² = 0%								
Test for overall effect: Z = 2.05 (P = 0.04)								
**7.2.2 post-lat female**								
Attenborough 2017	71.3	10	11	71.4	10.4	83	16.0%	-0.01 [-0.64, 0.62]
Hartley 2018	105.65	13.47	21	100.65	10.41	145	22.6%	0.46 [-0.00, 0.92]
**Subtotal (95% CI)**			**32**			**228**	**38.5%**	**0.28 [-0.17, 0.72]**
Heterogeneity: Tau² = 0.03; Chi² = 1.39, df = 1 (P = 0.24); I² = 28%								
Test for overall effect: Z = 1.21 (P = 0.23)								
**Total (95% CI)**			**125**			**1055**	**100.0%**	**-0.03 [-0.35, 0.29]**
Heterogeneity: Tau² = 0.06; Chi² = 7.56, df = 3 (P = 0.06); I² = 60%								
Test for overall effect: Z = 0.18 (P = 0.85)								
Test for subgroup differences: Chi² = 3.88, df = 1 (P = 0.05), I² = 74.2%								

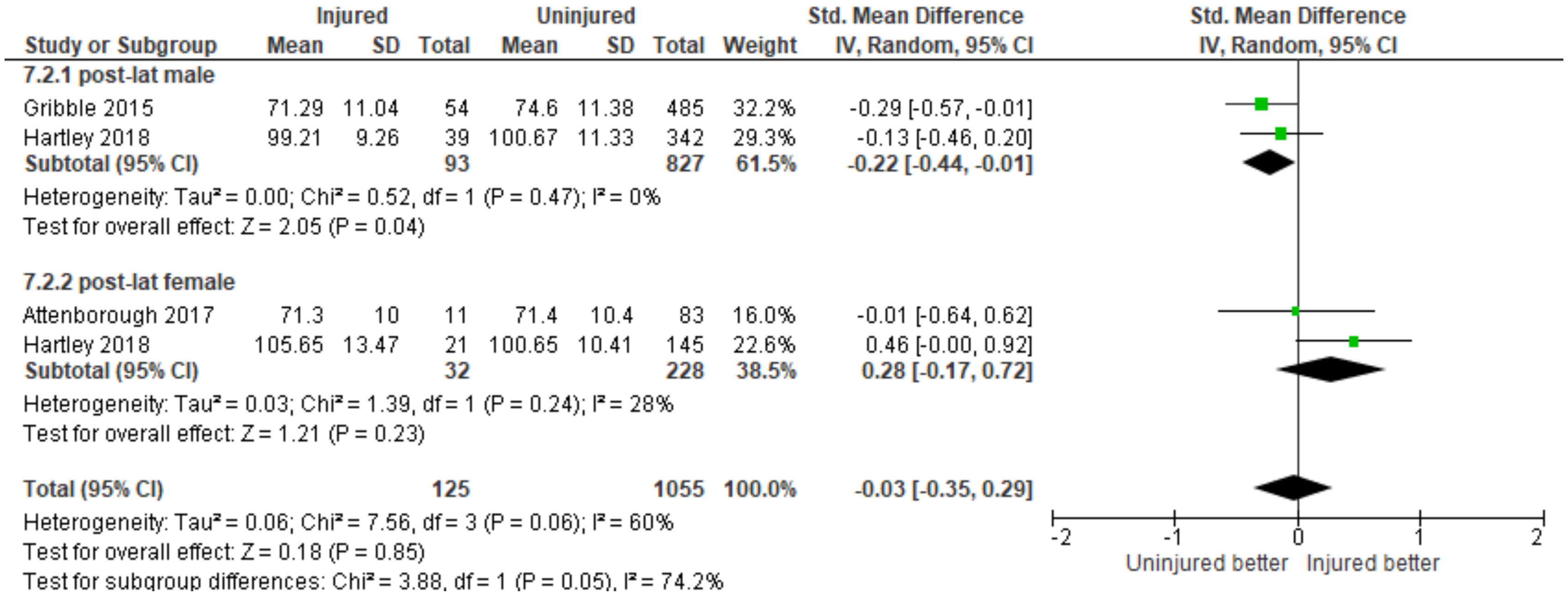

Supp Fig 15. Forest plot depicting the meta-analytical results for *posterior dynamic balance* as a risk factor for ankle sprain injury in males and females

Study or Subgroup	Injured Mean	Injured SD	Injured Total	Uninjured Mean	Uninjured SD	Uninjured Total	Weight	Std. Mean Difference IV, Random, 95% CI
**7.3.1 post-med male**								
Gribble 2015	84.35	11.91	54	85.76	11.03	485	35.4%	-0.13 [-0.41, 0.15]
Hartley 2018	106.37	8.06	39	105.63	10.25	342	30.5%	0.07 [-0.26, 0.40]
**Subtotal (95% CI)**			**93**			**827**	**65.9%**	**-0.04 [-0.26, 0.17]**
Heterogeneity: Tau² = 0.00; Chi² = 0.81, df = 1 (P = 0.37); I² = 0%								
Test for overall effect: Z = 0.39 (P = 0.70)								
**7.3.2 post-med female**								
Attenborough 2017	76.1	7.8	11	78.2	9.4	83	13.3%	-0.23 [-0.86, 0.40]
Hartley 2018	109.36	10.72	21	105.17	8.89	145	20.9%	0.46 [-0.00, 0.92]
**Subtotal (95% CI)**			**32**			**228**	**34.1%**	**0.15 [-0.51, 0.82]**
Heterogeneity: Tau² = 0.15; Chi² = 2.94, df = 1 (P = 0.09); I² = 66%								
Test for overall effect: Z = 0.44 (P = 0.66)								
**Total (95% CI)**			**125**			**1055**	**100.0%**	**0.04 [-0.22, 0.30]**
Heterogeneity: Tau² = 0.03; Chi² = 5.18, df = 3 (P = 0.16); I² = 42%								
Test for overall effect: Z = 0.32 (P = 0.75)								
Test for subgroup differences: Chi² = 0.30, df = 1 (P = 0.59), I² = 0%								

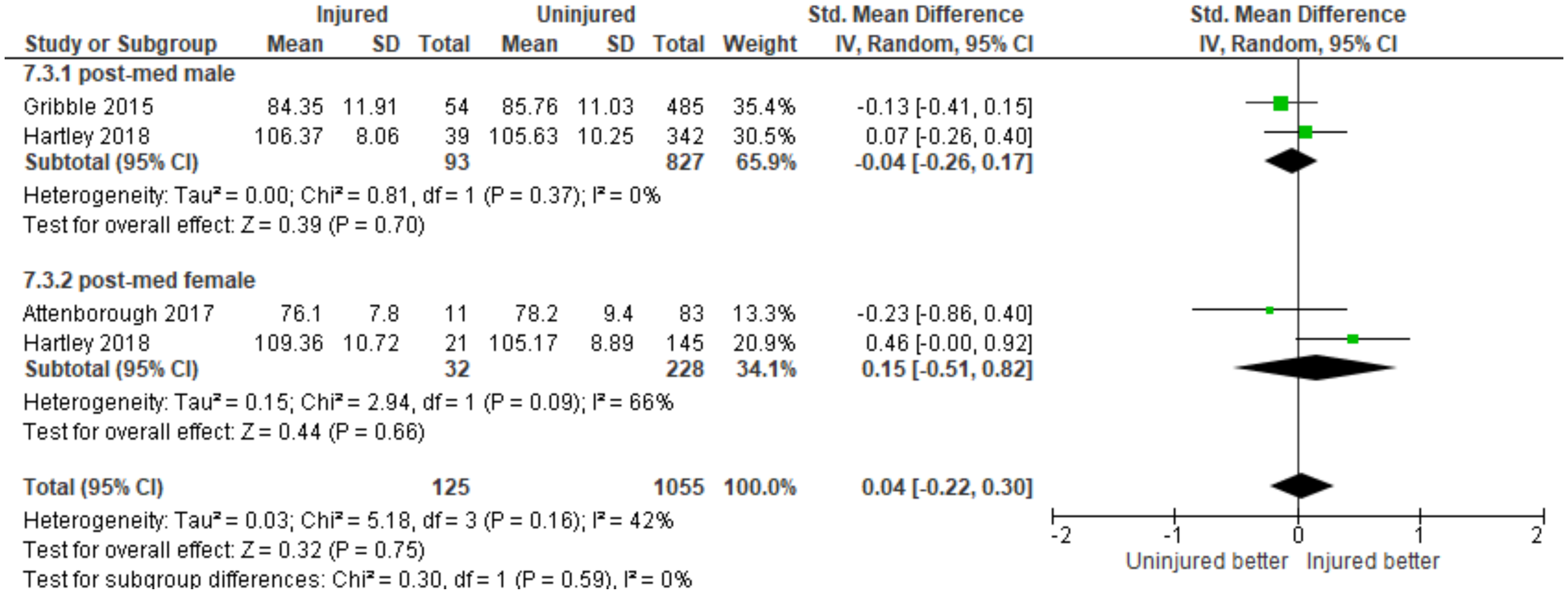

Supp Fig 16. Forest plot depicting the meta-analytical results for *posterior medial dynamic balance* as a risk factor for ankle sprain injury in males and females

	Injured			Uninjured				Std. Mean Difference
**Study or Subgroup**	**Mean**	**SD**	**Total**	**Mean**	**SD**	**Total**	**Weight**	**IV, Random, 95% CI**
**8.1.1 Dorsiflexion ROM male**								
Beynnon 2001	12.7	3.9	7	11.3	3.7	43	2.7%	0.37 [-0.43, 1.17]
Engebretsen 2010	10.1	5.3	38	10.4	7.3	827	16.4%	-0.04 [-0.37, 0.28]
Hartley 2018	8.19	3.24	39	8.51	3.6	342	15.8%	-0.09 [-0.42, 0.24]
Kawaguchi 2021	40.4	7.1	31	39.6	6.3	228	12.3%	0.12 [-0.25, 0.50]
Saki 2021	22.26	6.09	34	21.32	7.41	118	11.9%	0.13 [-0.25, 0.51]
Willems 2005a	32.88	6.06	44	34.85	7.59	197	16.2%	-0.27 [-0.60, 0.06]
**Subtotal (95% CI)**			**193**			**1755**	**75.4%**	**-0.03 [-0.18, 0.12]**
Heterogeneity: Tau² = 0.00; Chi² = 4.44, df = 5 (P = 0.49); I² = 0%								
Test for overall effect: Z = 0.40 (P = 0.69)								
**8.1.2 Dorsiflexion ROM female**								
Beynnon 2001	12.8	3.5	13	12.3	3.3	55	4.7%	0.15 [-0.46, 0.75]
Hartley 2018	9.44	2.79	21	9.06	3.36	145	8.3%	0.11 [-0.34, 0.57]
Willems 2005b	38.19	10.63	32	37.39	6.61	127	11.6%	0.11 [-0.28, 0.49]
**Subtotal (95% CI)**			**66**			**327**	**24.6%**	**0.12 [-0.15, 0.38]**
Heterogeneity: Tau² = 0.00; Chi² = 0.01, df = 2 (P = 0.99); I² = 0%								
Test for overall effect: Z = 0.86 (P = 0.39)								
**Total (95% CI)**			**259**			**2082**	**100.0%**	**0.01 [-0.13, 0.14]**
Heterogeneity: Tau² = 0.00; Chi² = 5.34, df = 8 (P = 0.72); I² = 0%								
Test for overall effect: Z = 0.08 (P = 0.94)								
Test for subgroup differences: Chi² = 0.89, df = 1 (P = 0.34), I² = 0%								

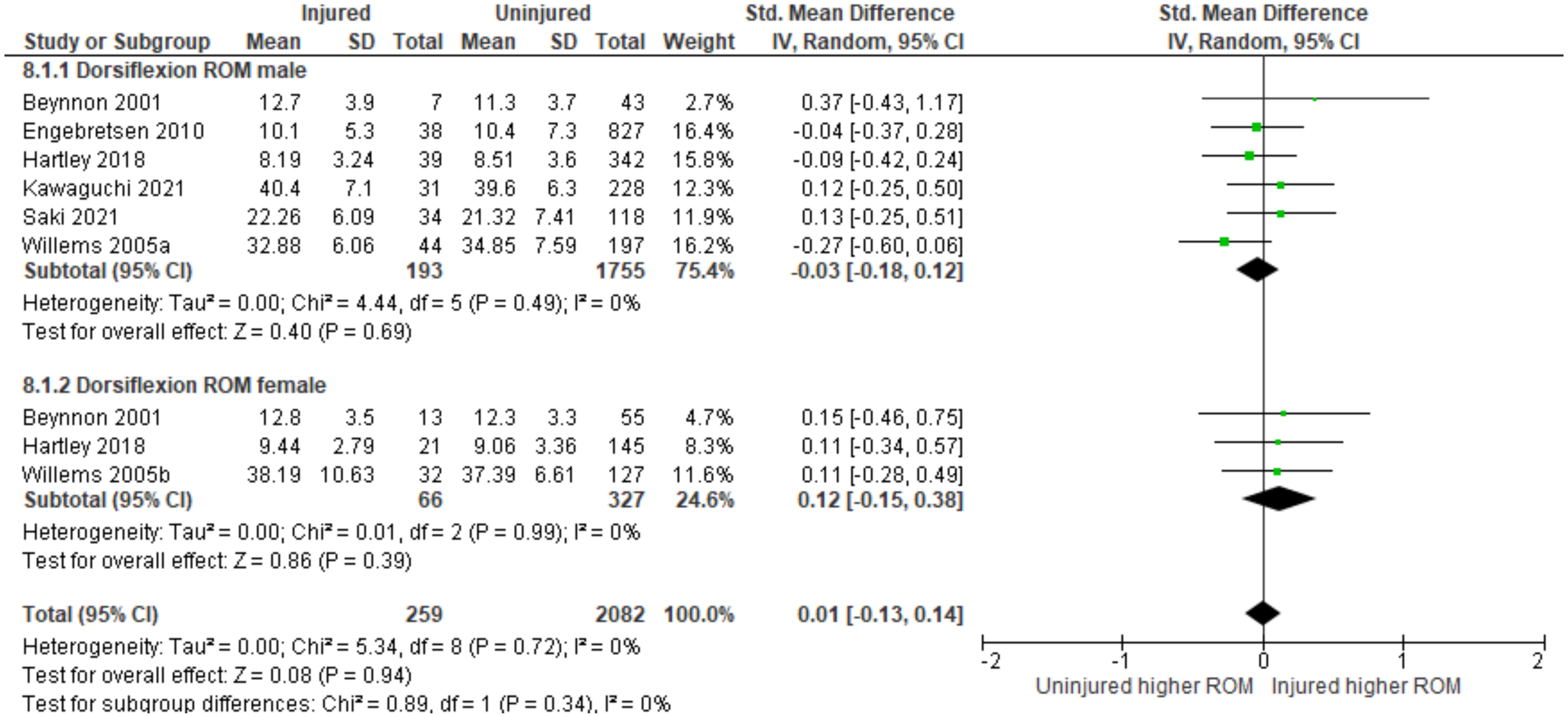

Supp Fig 17. Forest plot depicting the meta-analytical results for *dorsiflexion range of movement* as a risk factor for ankle sprain injury in males and females

Study or Subgroup	Injured Mean	Injured SD	Injured Total	Uninjured Mean	Uninjured SD	Uninjured Total	Weight	Std. Mean Difference IV, Random, 95% CI
**8.2.1 Plantarflexion ROM male**								
Saki 2021	25.24	7.21	34	29.22	7.44	118	32.0%	-0.54 [-0.92, -0.15]
Willems 2005a	52.03	8.64	44	52.39	8.29	197	36.0%	-0.04 [-0.37, 0.28]
**Subtotal (95% CI)**			**78**			**315**	**68.1%**	**-0.28 [-0.76, 0.20]**
Heterogeneity: Tau² = 0.09; Chi² = 3.64, df = 1 (P = 0.06); I² = 73%								
Test for overall effect: Z = 1.13 (P = 0.26)								
**8.2.2 Plantarflexion ROM female**								
Willems 2005b	58.92	6.51	32	58.34	8.48	127	31.9%	0.07 [-0.32, 0.46]
**Subtotal (95% CI)**			**32**			**127**	**31.9%**	**0.07 [-0.32, 0.46]**
Heterogeneity: Not applicable								
Test for overall effect: Z = 0.36 (P = 0.72)								
**Total (95% CI)**			**110**			**442**	**100.0%**	**-0.16 [-0.51, 0.19]**
Heterogeneity: Tau² = 0.06; Chi² = 5.49, df = 2 (P = 0.06); I² = 64%								
Test for overall effect: Z = 0.92 (P = 0.36)								
Test for subgroup differences: Chi² = 1.22, df = 1 (P = 0.27), I² = 18.2%								

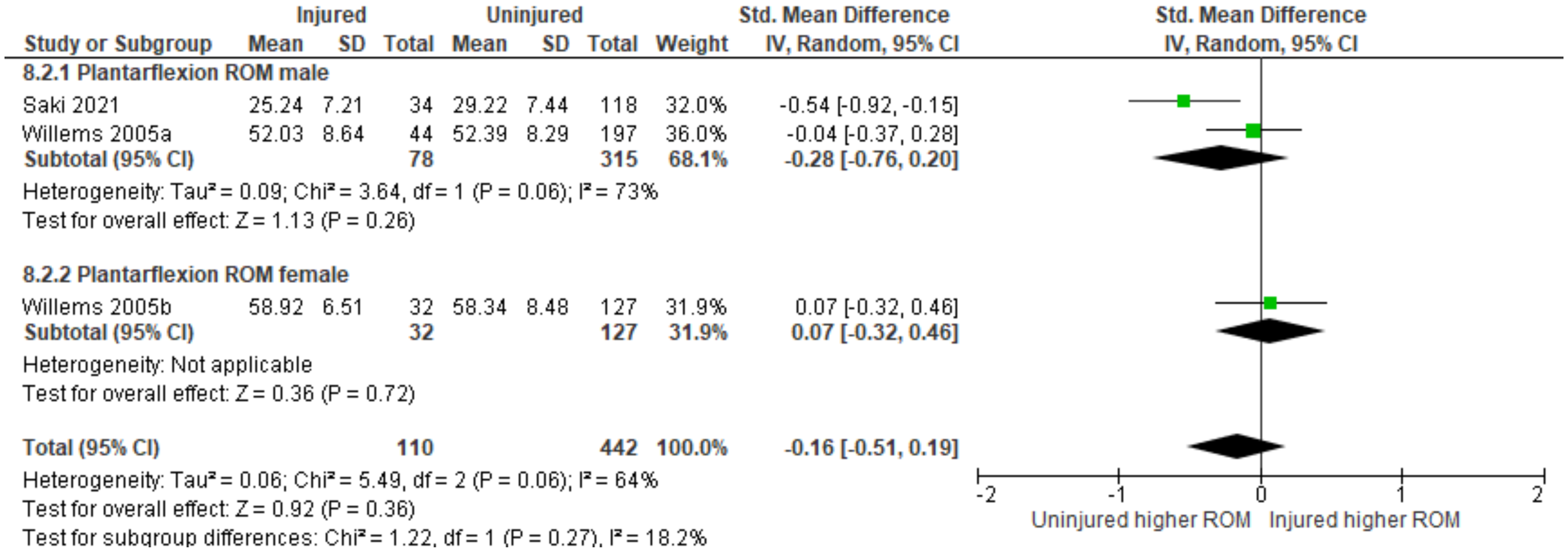

Supp Fig 18. Forest plot depicting the meta-analytical results for *plantarflexion range of movement* as a risk factor for ankle sprain injury in males and females

Study or Subgroup	Injured Mean	Injured SD	Injured Total	Uninjured Mean	Uninjured SD	Uninjured Total	Weight	Std. Mean Difference IV, Random, 95% CI
**8.3.1 Inversion ROM male**								
Beynnon 2001	15.3	3.6	7	18	5.9	43	7.6%	-0.47 [-1.27, 0.34]
Willems 2005a	19.41	8	44	18.9	6.49	197	46.2%	0.07 [-0.25, 0.40]
**Subtotal (95% CI)**			**51**			**240**	**53.8%**	**-0.07 [-0.54, 0.40]**
Heterogeneity: Tau² = 0.05; Chi² = 1.51, df = 1 (P = 0.22); I² = 34%								
Test for overall effect: Z = 0.28 (P = 0.78)								
**8.3.2 Inversion ROM female**								
Beynnon 2001	17.8	4.6	13	16.8	5.4	55	13.5%	0.19 [-0.42, 0.79]
Willems 2005b	20.69	7.3	32	21.75	8.25	127	32.8%	-0.13 [-0.52, 0.26]
**Subtotal (95% CI)**			**45**			**182**	**46.2%**	**-0.04 [-0.36, 0.29]**
Heterogeneity: Tau² = 0.00; Chi² = 0.75, df = 1 (P = 0.39); I² = 0%								
Test for overall effect: Z = 0.23 (P = 0.82)								
**Total (95% CI)**			**96**			**422**	**100.0%**	**-0.02 [-0.24, 0.20]**
Heterogeneity: Tau² = 0.00; Chi² = 2.29, df = 3 (P = 0.52); I² = 0%								
Test for overall effect: Z = 0.17 (P = 0.87)								
Test for subgroup differences: Chi² = 0.01, df = 1 (P = 0.92), I² = 0%								

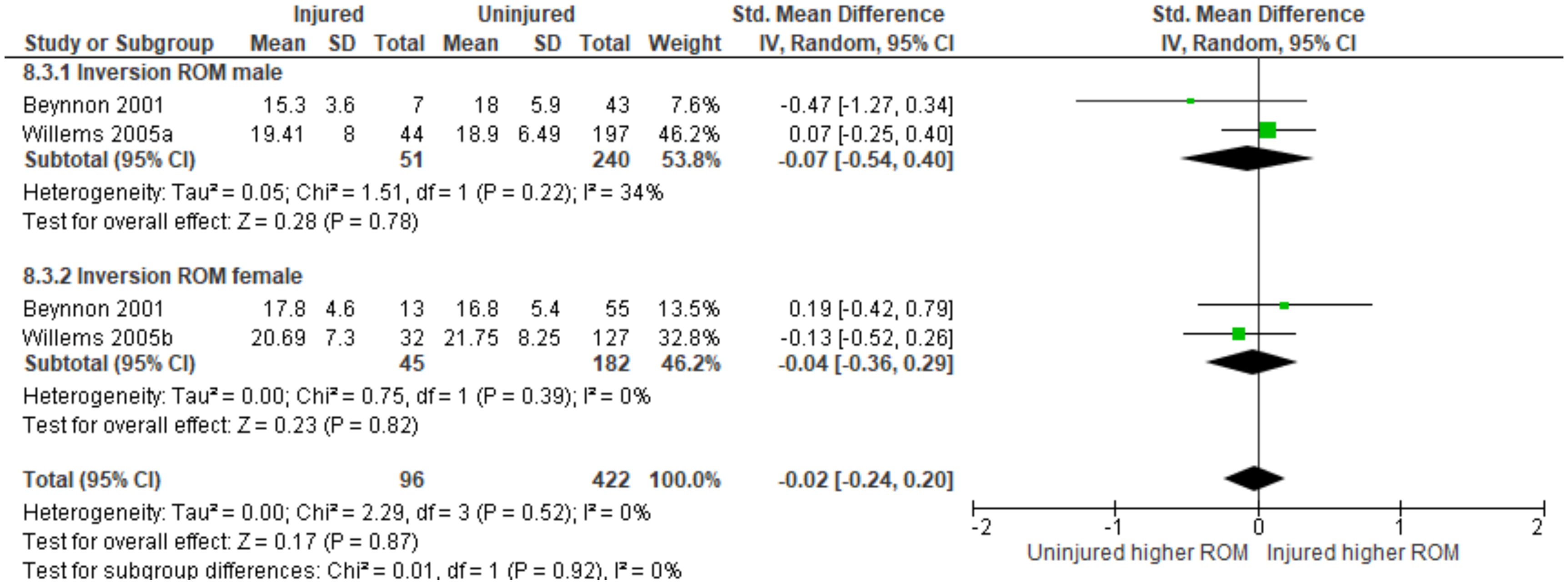

Supp Fig 19. Forest plot depicting the meta-analytical results for *inversion range of movement* as a risk factor for ankle sprain injury in males and females

Study or Subgroup	Injured Mean	Injured SD	Injured Total	Uninjured Mean	Uninjured SD	Uninjured Total	Weight	Std. Mean Difference IV, Random, 95% CI
**8.4.1 Eversion ROM male**								
Beynnon 2001	9	5.59	7	10.55	4.58	43	12.7%	-0.32 [-1.12, 0.48]
Willems 2005a	5.4	2.3	44	4.3	2.4	197	36.5%	0.46 [0.13, 0.79]
**Subtotal (95% CI)**			**51**			**240**	**49.2%**	**0.16 [-0.59, 0.91]**
Heterogeneity: Tau² = 0.21; Chi² = 3.14, df = 1 (P = 0.08); I² = 68%								
Test for overall effect: Z = 0.41 (P = 0.68)								
**8.4.2 Eversion ROM female**								
Beynnon 2001	6.1	2.6	13	5.2	1.8	55	19.0%	0.45 [-0.16, 1.06]
Willems 2005b	9.85	5.82	32	9.75	5.35	127	31.8%	0.02 [-0.37, 0.41]
**Subtotal (95% CI)**			**45**			**182**	**50.8%**	**0.17 [-0.24, 0.57]**
Heterogeneity: Tau² = 0.03; Chi² = 1.38, df = 1 (P = 0.24); I² = 28%								
Test for overall effect: Z = 0.82 (P = 0.41)								
**Total (95% CI)**			**96**			**422**	**100.0%**	**0.22 [-0.10, 0.54]**
Heterogeneity: Tau² = 0.05; Chi² = 5.32, df = 3 (P = 0.15); I² = 44%								
Test for overall effect: Z = 1.33 (P = 0.18)								
Test for subgroup differences: Chi² = 0.00, df = 1 (P = 0.98), I² = 0%								

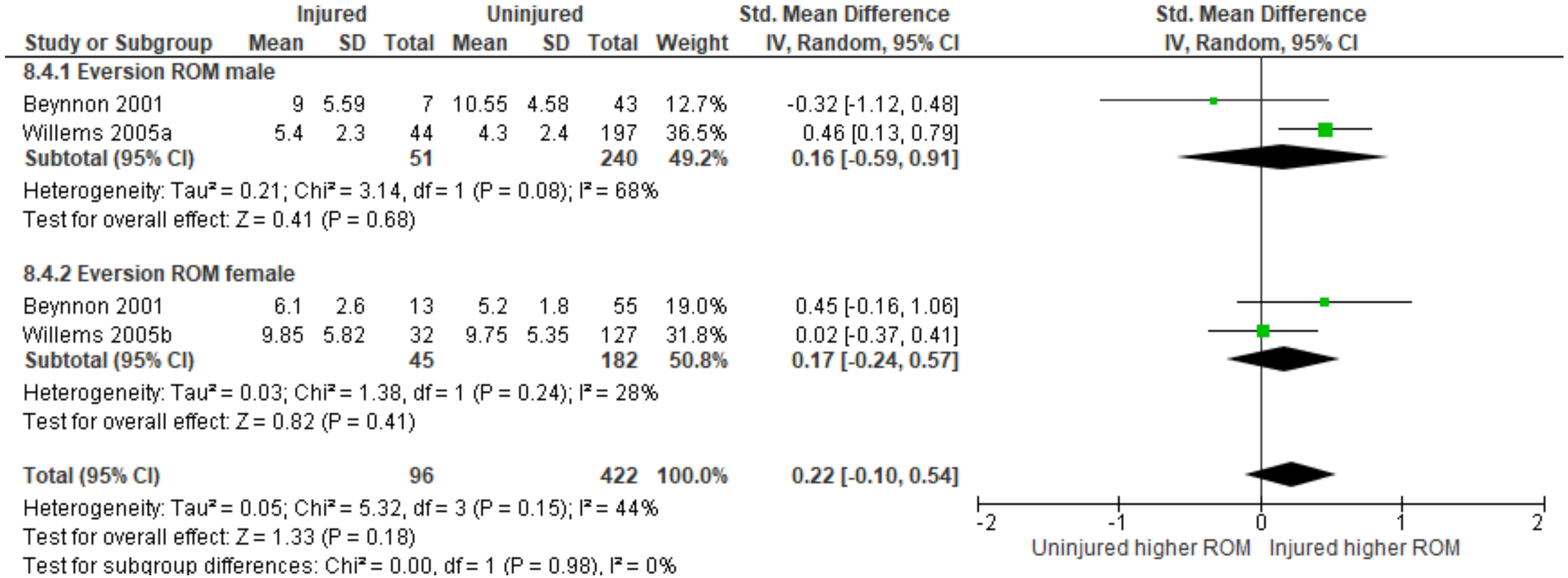

Supp Fig 20. Forest plot depicting the meta-analytical results for *eversion range of movement* as a risk factor for ankle sprain injury in males and females

Study or Subgroup	Injured Mean	Injured SD	Injured Total	Uninjured Mean	Uninjured SD	Uninjured Total	Weight	Std. Mean Difference IV, Random, 95% CI
**8.5.1 hip int rot ROM male**								
Kawaguchi 2021	36.4	6.6	31	35.4	8.5	228	32.1%	0.12 [-0.26, 0.50]
Willems 2005a	33.73	4.67	44	34.56	5.38	197	36.9%	-0.16 [-0.48, 0.17]
**Subtotal (95% CI)**			**75**			**425**	**69.0%**	**-0.03 [-0.30, 0.24]**
Heterogeneity: Tau² = 0.01; Chi² = 1.19, df = 1 (P = 0.27); I² = 16%								
Test for overall effect: Z = 0.25 (P = 0.80)								
**8.5.2 hip int rot ROM female**								
Willems 2005b	44.41	6.17	32	42.05	7.06	137	31.0%	0.34 [-0.05, 0.73]
**Subtotal (95% CI)**			**32**			**137**	**31.0%**	**0.34 [-0.05, 0.73]**
Heterogeneity: Not applicable								
Test for overall effect: Z = 1.73 (P = 0.08)								
**Total (95% CI)**			**107**			**562**	**100.0%**	**0.09 [-0.20, 0.37]**
Heterogeneity: Tau² = 0.03; Chi² = 3.80, df = 2 (P = 0.15); I² = 47%								
Test for overall effect: Z = 0.59 (P = 0.56)								
Test for subgroup differences: Chi² = 2.43, df = 1 (P = 0.12), I² = 58.8%								

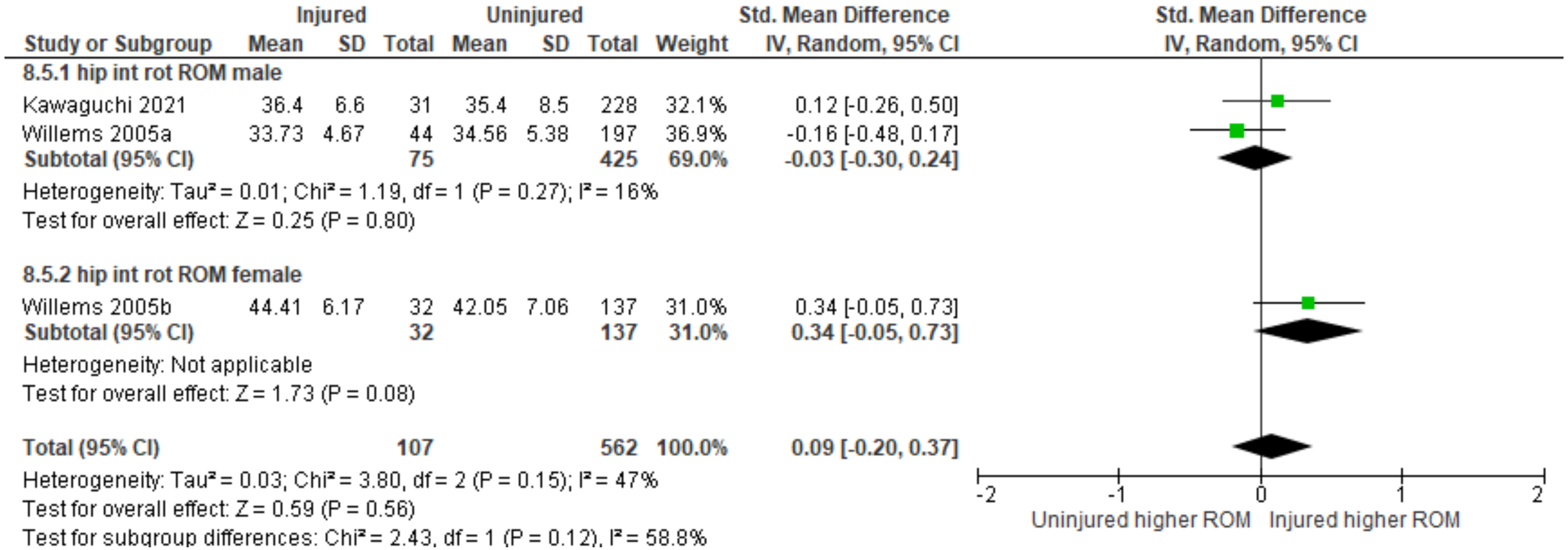

Supp Fig 21. Forest plot depicting the meta-analytical results for *hip internal rotation range of movement* as a risk factor for ankle sprain injury in males and females
